# Wild and cultivated comestible plant species in the Gulf of Mexico: phylogenetic patterns and convergence of type of use

**DOI:** 10.1093/aobpla/plad063

**Published:** 2023-09-01

**Authors:** Milton H Díaz-Toribio, J Arturo de-Nova, Eva María Piedra-Malagón, Diego F Angulo, Victoria Sosa

**Affiliations:** Jardín Botánico Francisco Javier Clavijero, Instituto de Ecología AC, Carretera antigua a Coatepec 351, El Haya, 91073 Xalapa, Veracruz, Mexico; Instituto de Investigación de Zonas Desérticas, Universidad Autónoma de San Luis Potosí, Altair 200, Colonia el Llano, 78377 San Luis Potosí, San Luis Potosí, Mexico; Jardín Botánico Francisco Javier Clavijero, Instituto de Ecología AC, Carretera antigua a Coatepec 351, El Haya, 91073 Xalapa, Veracruz, Mexico; Unidad de Recursos Naturales, Centro de Investigación Científica de Yucatán, Calle 43 no. 130, Chuburná de Hidalgo, 97205 Mérida, Yucatán, Mexico; Biología Evolutiva, Instituto de Ecología AC, Mexico, Carretera antigua a Coatepec 351, El Haya, 91073 Xalapa, Veracruz, Mexico

**Keywords:** condiment plants, food plants, hot nodes, Mesoamerica, phylogenetic patterns

## Abstract

Cross-cultural research on edible plants might include ecological and evolutionary perspectives to understand processes behind species selection and management. With a database of approximately 500 comestible plants of the Province of the Gulf of Mexico in Mesoamerica, phylogenetic analyses are conducted to identify convergence and phylogenetic signal of type of use and significant clustering in the resulting phylogenetic trees. Analyses considered type of management (wild/managed vs. cultivated), type of use (edible, condiment, for wrapping food) and organ utilized. Elevated phylogenetic diversity and signal are expected for wild comestible taxa, indicating that people are using lineages across the angiosperm tree for food, resulting in broadness in diet and use of their regional resources. Main results are: (i) condiment species were identified in groups with an elevated phylogenetic signal; (ii) hot nodes for lineages utilized for wrapping food were found in many monocot groups as well as in epiphytes of cloud forests with leathery leaves; (iii) edible taxa were identified with the highest significant clustering restricted to certain branches in the phylogeny; (iv) wild and cultivated edible plants belong to identical lineages with replacement of species, implying that same plant groups known for their comestible benefits are substituted by species distributed in the Province and (v) wild versus cultivated lineages for condiment are different. Most food species in the Province belong to four families, namely Fabaceae, Cactaceae, Solanaceae and Asparagaceae. Analyses discovered underutilized wild species in identical clades to managed/cultivated taxa that can be studied further to identify cultivation practices. Results suggest that people are utilizing different lineages in the angiosperm tree available locally, for particular uses, like condiment or for wrapping food. Evidence can be used to study further undervalued edible species closely related to the most common food taxa as well as for bioprospection of their nutritional content.

## Teaser

Phylogenetic signal of 500 comestible plant species in the Gulf of Mexico indicates that people are using lineages across the angiosperm tree for food, resulting in broadness in diet and use of their regional resources.

## Introduction

Mesoamerica is a region that has been considered a centre of agricultural origin where many edible plants have been domesticated. Among the most prominent are amaranth, avocado, beans, maize, chillies, papaya, pumpkin and vanilla, and these plants continue to be managed and widely cultivated (Vavilov 1926; Harlan 1971; Khouri *et al*. 2016; Pickersgill 2016; Pironon *et al*. 2020). Moreover, the peoples of Mesoamerica have been and remain engaged in diverse *in situ* agricultural practices involving wild and weed species to control the availability of useful plants, most of which are edible species (Bye 1993; Casas *et al.* 2007; Vibrans 2016). Perhaps the most remarkable comestible wild and weedy plants in this region are ‘quelites’, which are edible greens closely associated with cornfields, crops and agricultural farming systems of nutritional importance (Bye 1981; Vyeyra-Odilón and Vibrans 2001; Carvalho and Barata 2016). Further noteworthy examples in Mesoamerica are several flowers and fruits gathered in natural ecosystems forming part of the people’s diet such as yucca flowers and sugar apple fruit (Sotelo *et al*. 2007; Núñez-Colín *et al*. 2008; Pérez-Negrón *et al*. 2014; Mapes and Basurto 2016; Figueredo-Urbina *et al*. 2021). Moreover, the wide variety of Mesoamerican edible plants (wild, managed and cultivated) has contributed enormously to the great diversity of traditional Mexican cuisine, which is considered an intangible heritage of humanity. Therefore, due to the relevance of comestible plants in Mesoamerica, we determine whether phylogenetic patterns differ in wild/managed versus cultivated species as well as in type of use as wrapping, condiment or food in a biogeographic region within Mesoamerica: the Province of the Gulf of Mexico.

Humans have access to a broad spectrum of food plants, varying to some extent as a result of cultural and plant regional diversity (Prescott-Allen and Prescott-Allen 1990). Cross-cultural research on diversity in the plant diet of humans is a field that combines anthropology, history, sociology and ecological and evolutionary perspectives, in order to understand the processes behind species selection and management (Proches *et al*. 2008; Alburquerque *et al*. 2015). Furthermore, discovery of phylogenetic patterns in edible plants reveals crucial ecological practices and illustrates the breadth of human diets (Proches *et al.* 2008). Phylogenetic analyses comprising edible species can identify patterns of incidence of concurrent nodes in cladograms (Gartnaje *et al*. 2017; Molina-Venegas *et al*. 2020). Likewise, estimation of the phylogenetic diversity (PD) of edible plants within a region is useful for understanding the extent of human diets associated with ethnic groups (Van Wyk 2019; Molina-Venegas *et al.* 2020; Pironon *et al.* 2020; Cordero *et al*. 2021; León-Lobos *et al.* 2022).

**Figure 1.**
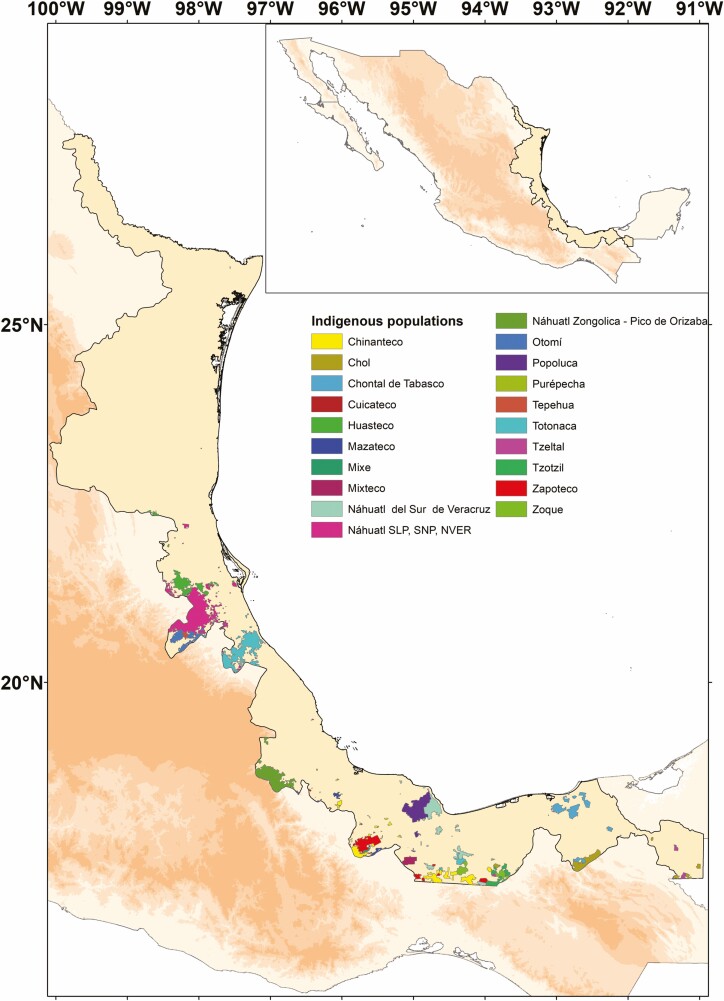
Limits of the study area, the biogeographic Province of the Gulf of Mexico. The different ethnic groups located within the Province are indicated.

Research on food plants around the world has found that different peoples prefer certain lineages and that associations exist between clades and edible usage (Van Wyk 2019; Molina-Venegas *et al.* 2020; Gaoue *et al.* 2021*a*, *b*; Cantwell-Jones *et al.* 2022). In the Province of the Gulf of Mexico, out of the approximately 500 food recorded plants, most species belong to four different families: Fabaceae, Cactaceae, Solanaceae and Asparagaceae (Piedra-Malagón *et al*. 2022). Moreover, this Province extends along the coastal plain of the Gulf of Mexico reaching altitudes up to 6000 m above sea level (see Piedra Malagón *et al.* 2022) (Fig. 1). The region with variable climate and forests from coniferous, tropical evergreen, deciduous and cloud forests to xerophilic scrubs and aquatic vegetation represents an extensive and remarkable variable region to study the phylogenetic patterns of the edible plants where about 15 ethnic groups inhabit this province (Challenger and Soberón 2008; Atlas de los Pueblos Indígenas de México 2020; Piedra Malagón *et al.* 2022).

Based on the database of comestible plant species of the Province of the Gulf of Mexico, phylogenetic analyses are conducted to identify convergence and phylogenetic signal of type of use and significant clustering in the resulting phylogenetic trees. We test several hypotheses that emerge regarding this collection of data: (i) same supported plant lineages will be identified in wild versus cultivated groups of taxa although containing different species; (ii) clustering of wild species utilized as condiment, as wrapping and as edible will be identified at restricted nodes of phylogenetic trees and (iii) wild species utilized as condiment and for wrapping food will be identified in different lineages compared to cultivated taxa. The results will determine whether the patterns in our database are supported in phylogenetic trees and discover new valuable lineages and underutilized species. Furthermore, they are important in terms of drawing attention to underestimated comestible plants, to understand selection of food plants in the Gulf of Mexico and diversification of diets in this region, as has been demonstrated previously for other regions (Sotelo *et al.* 2007; Ulian *et al*. 2020; Georgiadis 2022).

## Material and Methods

### Database

Wild and managed/cultivated vascular plants, classed as food (edible, condiment, or wrapping), considering plant organs utilized (roots/bulbs/rhizomes, stems, bark, wood, latex/resin, leaves, flowers, fruits, seeds) were considered (Piedra-Malagón *et al.* 2022). Information was based on fieldwork conducted as part of our project, the relevant published literature and data included with specimens deposited in the herbaria of this Province (Piedra-Malagón *et al.* 2022). The database was comprised of 473 species of vascular plants distributed in the Province of the Gulf of Mexico and was utilized to carry out analyses. The nomenclature was corrected from the initial number of the published database (487), based on World Flora Online (www.worldfloraonline.org) (Piedra-Malagón *et al.* 2022).

### Phylogeny reconstruction

A recently published mega-phylogeny known as ‘GBOTB’ was the basis on which a phylogeny was generated for the edible species recorded in the Province of the Gulf of Mexico. GBOTB had been constructed with 79 881 taxa in GenBank and using the backbone provided by Open Tree of Life (Smith and Brown 2018). This constitutes the most comprehensive and up-to-date time-calibrated species-level mega-phylogeny for seed plants. To build the phylogeny for the edible species, Phylomatic and BLADJ approaches (Webb *et al.* 2008) implemented in the V.PhyloMaker (scenario 3) were utilized (Jin and Qian 2019). V.PhyloMaker places any missing species at the basal node within a given genus and any missing genera at the basal node within their respective families. We pruned the phylogeny in order to retain only the species present in our database.

### Phylogenetic signal

To test phylogenetic signal, type of management, type of use and organ utilized were coded for every species and analyses were conducted based on the phylogenetic tree already constructed. Separate analyses were conducted for every type of use and management and for all uses. The method developed by Fritz and Purvis (2010) was selected to estimate phylogenetic dispersion (*D*) for discrete traits, with the R package ‘caper’ (Orme 2018). We performed 1000 permutations based on random patterns and these were compared to the observed phylogenetic pattern distributions for each food category and plant organ utilized in order to evaluate whether this value differed significantly from that expected with no (random) phylogenetic structure. A decrease in the values of *D* from 1 increases phylogenetic clumping in the binary trait; therefore, a *D* statistic value of 0 indicates that the trait is phylogenetically conserved (phylogenetic signal), as would be expected under a Brownian model of trait evolution, while a value of 1 suggests a random mode of evolution (no phylogenetic signal) (Fritz and Purvis 2010). If the observed *D* value is found between 0 and 1, but with a significant departure from 1 (random), this means the trait is non-random along the phylogeny. Otherwise, the trait is considered random. The phylogenetic signal in the studied characters was graphically illustrated using the ‘contmap’ function of the phytools package (Revell 2012) of R. All analyses were performed using R version 3.6.0 (R Core Team 2019).

### PD and clustering

 Phylogenetic diversity *sensu stricto* (PD), the mean pairwise phylogenetic distance (MPD) and the mean pairwise distance to the nearest taxon (MNTD) were estimated for every type of use (edible, condiment, wrapping) and for wild/cultivated species. PD and MNTD are terminal metrics reflecting phylogenetic structure that is dominant near the tip of the tree, while MPD considers basal branches of the phylogeny (Webb *et al.* 2008; Mazel *et al.* 2016). The standardized effect sizes of these metrics were determined to explore phylogenetic structure of type of use guilds since they test significant phylogenetic clustering at the full phylogeny, as well as at the basal and terminal branches (Webb *et al.* 2002; Mazel *et al.* 2016). Moreover, they quantify the relative excess (overdispersion) or deficit (clustering) in PD for a given set of species for each type of use relative to species pool in the full phylogeny. A negative standardized metric reflects a relative clustering of species while a positive standardized metric reflects a relative overdispersion of species (Mazel *et al*. 2016). Analyses were performed in Picante (Kembel *et al.* 2010) for R v.4.0.5 (R Core Development Team 2021). Significant phylogenetic clustering was detected when *P* values of the standardized metrics were below 0.05 and significant overdispersion when *P* values were above 0.95, equivalent to a standard effect size >1.96 or <-1.96 (Mazel *et al.* 2016).

To assess the degree of phylogenetic overlap between clusters of wild versus cultivated groups and groups according to type of use we explored phylogenetic beta diversity (PBD) using the PhyloSor index (Bryant *et al.* 2008) as a distance metric (see Molina-Venegas *et al*. 2020). PBD is defined as 1—PhyloSor index and can be decomposed into two additive components, namely ‘true’ phylogenetic turnover (hereafter ‘turnover’) and nestedness, which represent different aspects of beta diversity (Leprieur *et al.* 2012; Molina-Venegas *et al*. 2020). We evaluated if the observed turnover component of PBD was higher (ses.PBD > 1.96) than expected for a given comparison by computing SES scores for the phylogenetic turnover (ses.pß_sim_) measured as Simpson-derived pairwise phylogenetic dissimilarity. The nestedness-resultant phylogenetic dissimilarity was measured as the nestedness-fraction of Sorensen (ses.pß_sne_) derived pairwise phylogenetic dissimilarity, and for PBD (ses.pß_sor_), it was measured as Sorensen-derived pairwise phylogenetic dissimilarity (Molina-Venegas *et al.* 2020). Higher than expected values would indicate that the replacement involves deeper nodes of the phylogenetic tree (i.e. low phylogenetic overlap). For these analyses, we used the code ses.phylo.beta.pair provided by Molina-Venegas *et al.* (2020) and the package betapart (Baselga 2010; Baselga *et al.* 2018) for R v.4.0.5 (R Core Development Team 2021).

### Hot nodes and patterns of convergence of type of use

Plant groups with a significantly high number of edible species were identified through the search for ‘hot nodes’ in the phylogenetic tree. Analyses were conducted for (i) all edible plants in the Province of the Gulf of Mexico, (ii) all edible plants considering each food use type (edible, condiment, wrapping) and (iii) wild/cultivated species. For each use type, the number of comestible species descending from each node of the phylogeny was recorded and compared to a null distribution of values generated by shuffling trait values across the tips of the phylogeny 999 times (Saslis-Lagoudakis *et al.* 2011; Molina-Venegas *et al.* 2020). For a nominal alpha of 5 %, the richness of edible plants in clade I will be higher than expected for the given null model if the corresponding SES score is >1.96. We only evaluated those clades that included 10 species or more, since previous studies have documented unacceptable rates of statistical errors for smaller lineages (Parra *et al.* 2010). The computer code to conduct the hot node analysis was provided by Molina-Venegas *et al.* (2020) and the analysis was performed in R v.4.0.5 (R Core Development Team 2021).

## Results

### Phylogenetic signal

Results of the analyses to determine phylogenetic signal using the D statistics test are presented in Table 1. These were conducted for all comestible species in the Province of the Gulf of Mexico (cultivated/wild-managed species), as well as separately for cultivated and managed/wild species, taking into account the type of use (edible, condiment, wrapping) and particular organ utilized (root/rhizome/bulb, stem, bark wood, leaf, flowers, fruits, seeds, resin/latex). Evidence of phylogenetic signal was determined in all edible plants, considering food use type (Table 1) (i) edible (*D* = 0.37, *P*_rand_ = <0.057), (ii) condiment (*D* = 0.47, *P*_rand_ = <0.004) and (iii) wrapping (*D* = 0.25, *P*_rand_ = <0.17). The only one of these categories that presented phylogenetic significance was condiment. Evidence of significant phylogenetic signal was detected in three plant organs utilized (root/rhizome/bulb, leaf and seeds). Phylogenetic signal in roots or bulbs (*D* = 0.43, *P*_rand_ = <0.031, Table 1) was found in families such as Smilacaceae (six spp.), Euphorbiaceae (four spp.) and Araceae (three spp.). Phylogenetic signal in stems (*D* = 0.56, *P*_rand_ = <0.001, Table 1) was related to clades formed by families such as Cactaceae (eight spp.), Piperaceae (six spp.) and Cucurbitaceae (four spp.). Phylogenetic signal for leaves (*D* = 0.37, *P*_rand_ = <0.002, Table 1) was found in families such as Asparagaceae (11 spp.), Amaranthaceae, Leguminosae and Piperaceae (7 spp.), Asteraceae and Solanaceae (6 spp.). Phylogenetic signal in flowers (*D* = 0.37, *P*_rand_ = <0.002, Table 1) was found in species of families such as Asparagaceae (21 spp.), Leguminosae (20 spp.), Arecaceae (9 spp.) and Cucurbitaceae (6 spp.). Phylogenetic signal in fruits (*D* = 0.02, *P*_rand_ = <0.593, Table 1) was found in species of families such as Leguminosae (32 spp.), Solanaceae (30 spp.), Cactaceae (24 spp.), Sapotaceae (14 spp.), Annonaceae (12 spp.), and Araceae, Myrtaceae and Rosaceae (11 spp.). Phylogenetic signal in seeds (*D* = 0.39, *P*_rand_ = <0.011, Table 1) was found in species of families such as Leguminosae (13 spp.), Malvaceae (9 spp.), Cucurbitaceae (6 spp.) and Amaranthaceae, Arecaceae and Juglandaceae, with 4 species per family. Phylogenetic signal in resin or latex (*D* = 0.31, *P*_rand_ = <0.37, Table 1) was found in species of the families Sapotaceae (3 spp.) and Leguminosae (10 spp.). In the case of bark (*D* = -0.06, *P*_rand_ = 0.57, Table 1) and wood (*D* = -0.47, *P*_rand_ = 0.63, Table 1), the phylogenetic signal was not significant. For analyses carried out considering only wild and exclusively cultivated taxa, the results for cultivated species showed that only the condiment food type presented a strong phylogenetic signal (*D* = 0.87 *P*_rand_ = 0.015, Table 1), with the use of stems found to be significant (*D* = 1.04, *P*_rand_ = 0.002, Table 1). For wild species, the uses of stems (*D* = 0.56, *P*_rand_ = 0.002, Table 1), leaves *D* = 0.42, *P*_rand_ = 0.002) and seeds (*D* = 0.41, *P*_rand_ = 0.011) were significant.

**Table 1.** Results of analyses utilizing the D statistics test to identify phylogenetic signal among cultivated and wild species in the edible plants of the Province of the Gulf of Mexico. Type of use and plant organ used were considered. Significant values for *D* and *P* are presented in bold.

		*D*	*p*
Cultivated + Wild	Edible	0.3748	0.057
	Condiment	**0.4794**	**0.004**
	Wrapping	0.2596	0.17
	Root/rhizome/bulb	**0.4363**	**0.031**
	Stem	**0.5627**	**0.001**
	Bark	-0.0648	0.57
	Wood	-0.4793	0.63
	Leaf	**0.3725**	**0.002**
	Flowers	0.1627	0.12
	Fruits	0.0244	0.593
	Seeds	**0.3949**	**0.011**
	Resin/latex	0.314	0.378
Cultivated	Edible	-0.1168	0.598
	Condiment	**0.8709**	**0.015**
	Wrapping	0.5567	0.277
	Root/rhizome/bulb	0.8858	0.215
	Stem	**1.0496**	**0.002**
	Bark	–	–
	Wood	–	–
	Leaf	0.3596	0.111
	Flowers	-0.0197	0.512
	Fruits	-0.4668	0.945
	Seeds	0.2859	0.184
	Resin/latex	–	–
Wild	Edible	0.3388	0.08
	Condiment	0.2661	0.098
	Wrapping	0.0702	0.428
	Root/rhizome/bulb	0.4148	0.051
	Stem	**0.5603**	**0.002**
	Bark	-0.1823	0.638
	Wood	-1.9851	0.668
	Leaf	**0.4288**	**0.002**
	Flowers	0.1277	0.222
	Fruits	-0.0035	0.514
	Seeds	**0.4137**	**0.011**
	Resin/latex	0.2194	0.429

### PD and phylogenetic clustering

**Figure 2.**
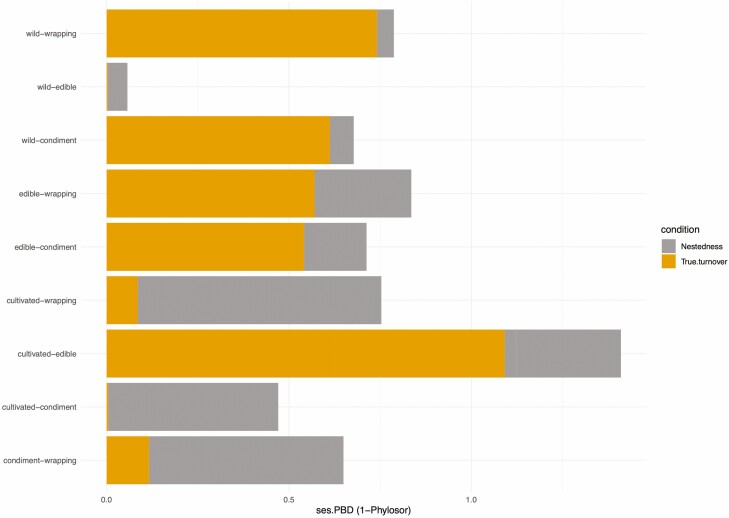
Graph displaying ‘true’ turnover and nestedness additive components of PBD between of the wild versus cultivated species as well as type of use of comestible species in the Province of the Gulf of Mexico. SES scores >1.96 are indicated with an asterisk.

The edible type of use was identified with the highest PD; in contrast, condiment had the highest MPD while wrapping the highest MNTD (Table 2). Standardized, ses.PD values indicate phylogenetic clustering for the type of food (wrapping, condiment, edible) with negative and significative values (*P* < 0.05). Basal phylogenetic clustering was detected only in wrapping (ses.MPD *P* values <0.05), and terminal phylogenetic clustering in condiment and wrapping (ses.MNTD *P* values <0.05) (Table 2). Phylogenetic metrics in wild taxa were higher in comparison with cultivated species, showing significant overdispersion in the entire phylogeny (positive values of PD; *P* > 0.95). Phylogenetic dissimilarities among type of use, estimated by PBD analyses were higher than expected (>0.64) with a true turnover component in edible versus condiment (0.54) and edible versus wrapping (0.57) comparisons (Table 3). Scores for ses.pßsim and ses.pßsor were >1.96 for edible versus condiment, edible versus wrapping and condiment versus wrapping, indicating that the replacement of species involves deeper nodes of the phylogenetic tree with significantly low overlap between these comparisons. In contrast, the nestedness component was higher (0.82) in condiment versus wrapping indicating high phylogenetic overlap between them (Table 3). Comparisons between type of use versus wild or cultivated indicate a higher true turnover component in edible versus cultivated (0.62), condiment versus wild (0.61) and wrapping versus wild (0.74) Fig. 2 displays these results, indicating whether significant nestedness versus true turnover occurred in the phylogenetic trees. Nestedness and true turnover for the comparison between wild versus cultivated taxa found that species are nested in the same lineages although they were different taxa (Fig. 2). For species utilized for wrapping food and as condiment they were not nested suggesting that they belong to different lineages (Fig. 2).

**Table 2.** Species richness (SR), phylogenetic diversity (PD), mean pairwise phylogenetic distance (MPD), mean pairwise distance to the nearest taxon (MNTD) and significant values ‘*P*’ estimated for the comestible plant species in the Province of the Gulf of Mexico. Standardized metrics are included as well (ses.PD, ses.MPD, ses.MNDT). Significant *P* values are marked with an asterisk.

Type of use	SR	PD	MPD	MNTD	ses.PD	*P*	ses.MPD	*P*	ses.MNDT	*P*
Edible	437	16 777	254	47.22	-2.1634	0.017*	-0.334	0.305	-1.190464	0.103
Condiment	51	3519.5	257	75.604	-2.7337	0.003*	0.72212	0.746	-3.235228	0.001*
Wrapping	26	2115.5	228	101.89	-2.781	0.003*	-1.0441	0.024*	-2.407228	0.006*
Cultivated	55	3578.5	253	83.382	-3.4877	0.001*	0.26812	0.565	-2.525447	0.004*
Wild	416	16 876	253	50.268	1.5522	0.950*	-0.9136	0.127	0.787675	0.785

**Table 3.** PhyloSor index for estimating turnover and nestedness additive components of the phylogenetic beta diversity (PBD) for the edible species of the Province of the Gulf of Mexico. Comparisons are indicated between wild versus edible, type of use (edible, condiment, wrapping). Standardized metrics (SES) scores >|1.96| are indicated with an asterisk.

Comparisons type of use and management	Nestedness (pß_sne)_	True turnover (pß_sim)_	Overall (pß_sim)_	ses.pß_sne_	ses.pß_sim_	ses.pß_sor_
Edible versus condiment	0.17	0.54	0.71	0.21	2.35*	3.44*
Edible versus wrapping	0.26	0.57	0.83	-1.49	2.91*	3.53*
Condiment versus wrapping	0.53	0.11	0.64	-0.69	2.03*	2.25*
Edible versus cultivated	0.03	0.62	0.65	2.64*	1.61	3.55*
Edible versus wild	0.05	0.002	0.05	-1.70	1.21	-0.26
Condiment versus cultivated	0.46	0.004	0.47	-1.02	0.28	-0.17
Condiment versus wild	0.06	0.61	0.67	2.56*	-0.17	2.87*
Wrapping versus cultivated	0.66	0.08	0.75	-1.83	3.77*	3.96*
Wrapping versus wild	0.04	0.74	0.78	1.84	0.46	2.82*

### Hot nodes and convergence of type of use

**Figure 3.**
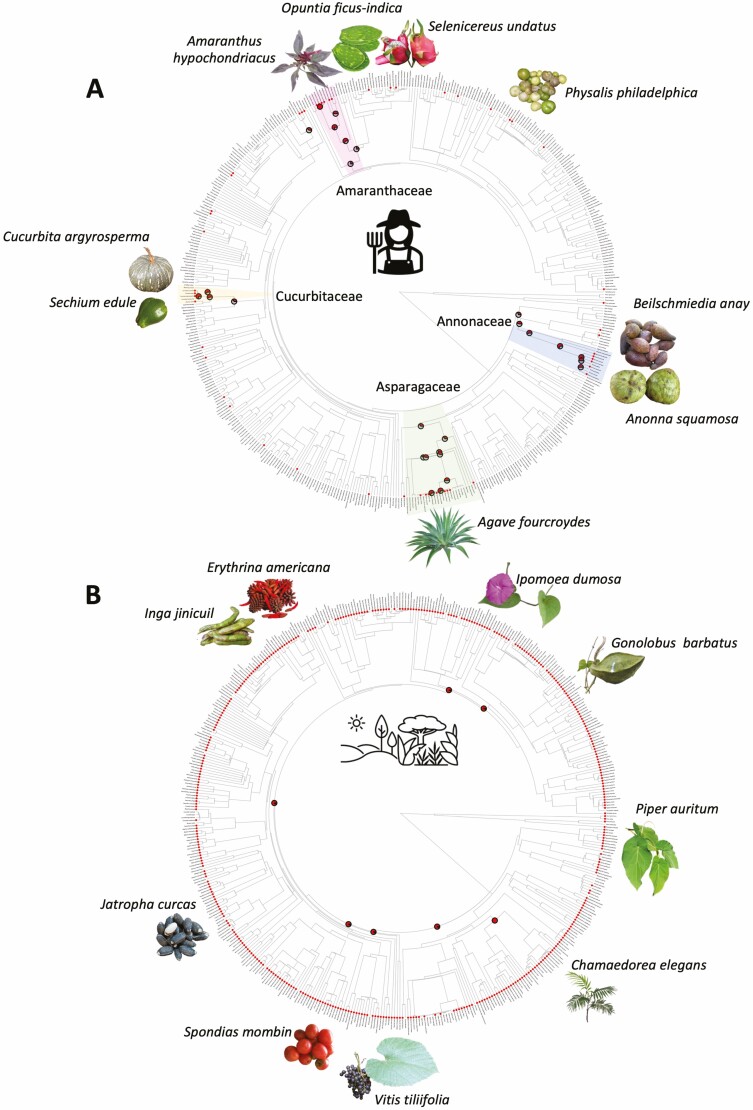
Phylogenetic trees displaying ‘hot nodes’ of analyses based on the comestible species in the Province of the Gulf of Mexico. (A) Tree illustrating studied edible plant species of the Province. (B) Tree displaying nodes for wild/incipient management edible plant taxa.

**Figure 4.**
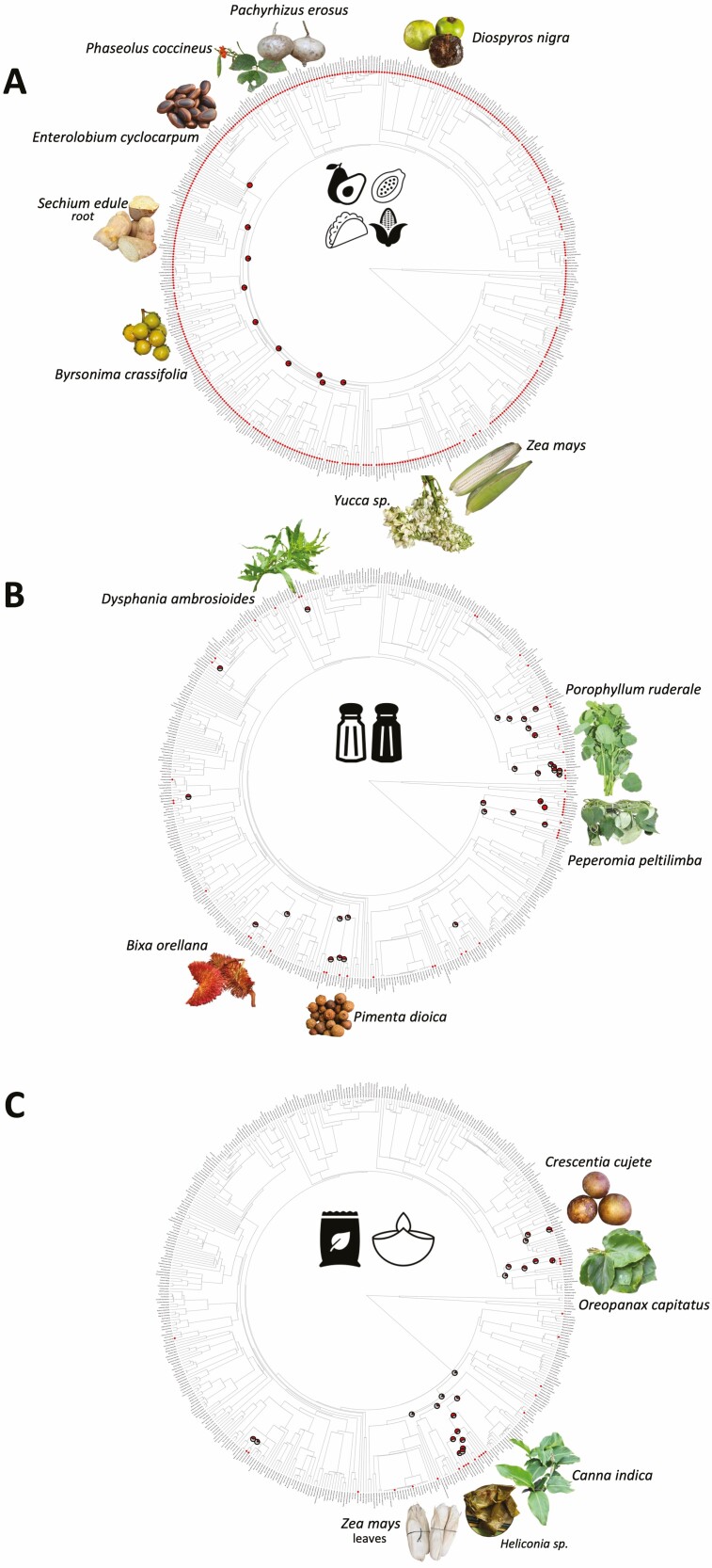
Phylogenetic trees displaying ‘hot nodes’ for the comestible type of use (edible, condiment, wrapping) based on the edible species in the Province of the Gulf of Mexico. (A) ‘Hot nodes’ for plants eaten raw or cooked. (B) ‘Hot nodes’ for plants utilized as condiments. (C) ‘Hot nodes’ for plants utilized for food wrapping.

The results of hot nodes revealed several important hot nodes grouping some lineages with a clear pattern of convergence in use (Fig. 3A). These include the node of Magnoliids, which comprises lineages such as Annonaceae (*Annona*: 5 spp., *Mosannona*) and several Piperaceae (*Piper* spp., *Peperomia*) and Lauraceae (*Persea* spp., *Beilschmiedia* sp.). A second node was identified in the order Caryophyllales, which includes species of Amaranthaceae (*Amaranthus* spp., *Atriplex* sp., *Chenopodium* sp., *Dysphania* sp.) and Cactaceae (*Myrtillocactus* sp., *Opuntia* spp., *Selenicereus* sp., *Stenocereus* sp.). An important node included taxa in Fabaceae. In addition, a hot node was determined in a clade in Cucurbitaceae comprising species of *Cucurbita* and *Sechium*, as well as a clade in Asparagales comprising *Agave* spp. and *Yucca* spp. For the analyses that exclusively considered wild/cultivated species, seven ‘hot nodes’ were evidenced; however, the most significant of these was found in the Monocots (Fig. 3B). This node comprised species utilized for food wrapping in the order Zingiberales: Cannaceae, Heliconiaceae and Marantaceae, as well as in order Arecales, in which several species of *Chamaedorea* present edible inflorescences. Regarding use type, edible species were scattered throughout the phylogenetic tree with hot nodes including many taxa in several lineages (Fig. 4A). For the phylogenetic tree presenting species used as condiments (Fig. 4B), two important hot nodes were determined. The first featured species in Piperaceae (*Piper* spp., *Peperomia* spp.), with a sister group comprising Lauraceae (*Licaria* sp., *Persea* spp.), and the second included several species in Asteraceae (*Tagetes* spp., *Dahlia* sp., *Porophyllum* sp.). There were also some small hot nodes; one of which included *Prosopis* spp. in the Fabaceae and *Dysphania* spp. in the Amaranthaceae. The phylogenetic tree comprising the species utilized for wrapping food presented two main hot nodes (Fig. 3C); one grouped species in *Oreopanax* in the Araliaceae and the other included lineages in the Monocots. *Zea mays, Calathea* sp., *Renealmia* sp., *Stromanthe* sp., *Heliconia* spp., *Canna* sp. were also identified in these lineages.

## Discussion

### Phylogenetic analyses and hot nodes

**Figure 5.**
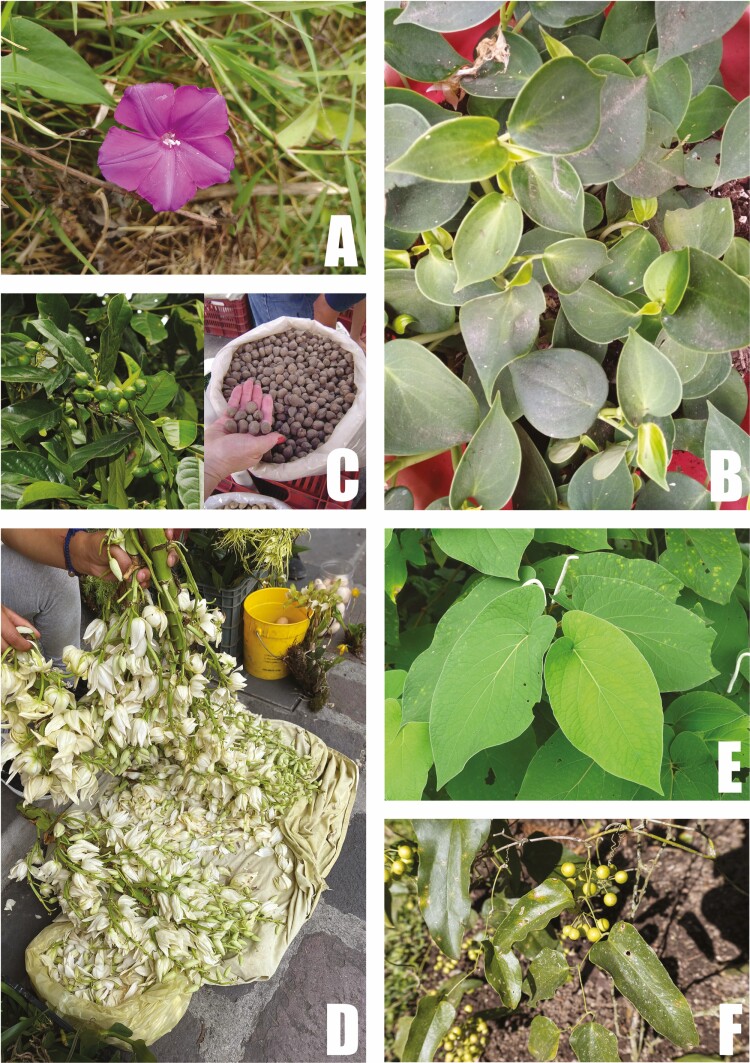
Remarkable edible plant species is utilized in the Province of the Gulf of Mexico. (A) *Ipomoea dumosa*, known as xonequi or xonegui, an edible green or quelite. (B) Leaves of *Peperomia peltilimba*, known as cilantro del monte, is utilized as a condiment. (C) Edible fruits of *Oecopetalum mexicanum*, known as cachichín. (D) Inflorescences of *Yucca* sp., the flowers of which are eaten by removing the stamens and added to soups. (E) *Piper auritum*, known as acuyo, is added to food as a condiment. (F) *Smilax aristolochiifolia*, known as ‘zarzaparrilla’ is the regional species utilized for producing root beer from rhizomes.

Species utilized as condiment were identified in lineages with elevated support and in significant hot nodes. Two groups stand out: Piperaceae and Asteraceae (see Fig. 5). The flavour of Piperaceae species is coriander-like and they have been used as condiments in many dishes since pre-Hispanic times (Picó and Nuez 2000; Lascurain-Rangel *et al.* 2022). Species are epiphytes common to cloud forests of the central area of the Province of the Gulf of Mexico (Vergara-Rodríguez *et al.* 2017). In addition, a third hot node lies in the Lamiaceae, in which several species of *Salvia* formed a group. Use of these taxa for providing a sage-like flavour to food has been recorded since pre-Hispanic times (Picó and Nuez 2000).

Furthermore, it is worth mentioning that hot nodes for lineages utilized for wrapping food were found in many monocot groups. Broad-leaved species in *Heliconia*, *Canna* and *Maranta* are utilized for wrapping tamales (Lascurain *et al*. 2017). Tamales are made of maize dough (sweet, salty or sour) with raisins or vegetables or meat with chilli wrapped with these leaves (Lascurain *et al*. 2017). In addition, another lineage for covering tamales with ample and leathery leaves in *Oreopanax* spp. of the Araliaceae was identified in a hot node (Lascurain *et al.* 2017). All these species possess similar anatomical characteristics featuring leathery leaves, highlighting the relevance of phenotypical convergence in human selection (Hawkins and Teixidor-Toneau 2017).

Species in lineages in cultivated and weedy/wild plants in which the stems and leaves are edible greens, the ‘quelites’, widely consumed in Mexico, were identified with elevated phylogenetic signal as well (Bye 1981; Linares *et al.* 2017). In the order Caryophyllales, the most important sub-node included quelites in genera such as *Amaranthus*. Amaranth is known as a pseudocereal and has been widely utilized in central Mexico since pre-Hispanic times (Marx 1977); however, its leaves, stems and inflorescences are eaten as quelites prepared in many dishes (Linares *et al.* 2017). Fruit species in the cucurbits *Sechium* and *Cucurbita* spp. and eight species of cherimolas or sugar apples, *Annona* spp. were identified in hot nodes in different lineages. These taxa have been amply recorded for several regions in Mesoamerica (Basurto-Peña *et al.* 2003; Escobedo-López *et al*. 2019).

Most of the hot nodes identified in the phylogeny coincided with lineages in the most diverse families identified in the Province of the Gulf of Mexico as comestible: Fabaceae, Cactaceae and Asparagaceae (Piedra-Malagón *et al.* 2022). Furthermore, approximately 12 % of the species recorded in this Province are cultivated while approximately 88 % are collected in the wild or under incipient management (Piedra-Malagón *et al.* 2022).

### PD and clustering

Regarding type of use, species in lineages classed as edible were identified with the highest PD, they represent the majority (97 %) of species and belong to diverse lineages thus reflecting evolutionary heterogeneity. It has been documented that the diversity of edible plants is related to biocultural processes as well as ecological variation (Carvalho and Barata 2016; Murphy and Fuller 2016). In the Province of the Gulf of Mexico, 15 ethnic groups and variation in vegetation exists; therefore, it was expected that many plant groups are eaten or utilized as condiment or for wrapping food.

Wild comestible taxa were identified with the highest significant clustering (MPD) restricted to certain branches in the phylogeny. Same lineages were identified again like in previous analyses. Species in the order Caryophyllales and species in *Annona* were grouped in clades of terminal branches of the phylogenetic trees. In Caryophyllales, different lineages in Cactaceae known for their edible fruits, such as pitahayas (*Selenicereus* spp.) and prickly pears (*Opuntia* spp.) (Ramírez-Rodríguez *et al.* 2020), or in Cucurbitaceae, with many edible fruits such as chayote (*Sechium edule*) or pumpkin (*Cucurbita* spp.) (Segura *et al*. 2018) were clustered in nodes with elevated MNTDs values. It is worth mentioning that species utilized for wrapping food and for condiment displayed high MNTDs as well, indicating basal or terminal clustering in cladograms. Furthermore, analyses that identify turnover and nestedness of clustering confirm these results. For wild species utilized as wrapping and for condiment, turnover is evident, meaning that lineages between species with this type of use are not shared between wild and cultivated clusters.

Phylogenetic dissimilarities quantified by PBD displayed significantly deep phylogenetic true turnover (i.e. low phylogenetic overlap) between edible lineages versus condiment lineages and for groups for wrapping food. Interestingly, true turnover in comparisons with managed plants was only detected in the edible category. We consider this pattern, in addition to phylogenetic clustering, as evidence of similar uses for plants included in certain nodes of the phylogeny.

### Implications of phylogenetic results

Phylogenetic analyses carried out with the approximately 500 comestible plant species in this study, helped to uncover ecological processes and selection by peoples in the Province of the Gulf of Mexico. Elevated PD determined in analyses including all species (wild/managed + cultivated) indicates that peoples are using lineages across the angiosperm tree for food, suggesting ample breadth in the people’s diet (Proches *et al*. 2008). Furthermore, species utilized for food vary according to culture, time and region (Prescott-Allen and Prescott-Allen 1990). Our study identified several lineages scattered in the angiosperm tree used for food that are distinctive of certain ecosystems in the Province of the Gulf of Mexico, such as cloud forests characterized by abundant epiphytes (Hietz and Hietz-Seifert 1995). Species in epiphytic genera, such as *Peperomia*, are utilized as condiment (Lascurain-Rangel *et al.* 2022), or like *Oreopanax*, a distant group in the phylogeny, which leaves are collected in the field for wrapping tamales (Lascurain *et al.* 2017). These results indicate that people are utilizing different lineages in the angiosperm tree available locally, for particular uses, like condiment or for wrapping food; moreover, phylogenetic metrics support these groupings depicted from the database. Convergence of type of use in lineages suggests that taxa possess either similar nutritional or flavouring compounds or morphological attributes in leaves for wrapping food.

Furthermore, true turnover and nestedness analyses for wild versus cultivated food species determined that different species are nested in the same lineages. As mentioned, most food species in the Province of the Gulf of Mexico belong to four families such as Fabaceae, Cactaceae, Solanaceae and Asparagaceae (Piedra-Malagón *et al.* 2022). They are well documented around the world for their comestible use (Hedrick 1972). Interestingly cultivated and wild species belong to these same lineages in this Province; however, composition of groups varies. Thus, results here discovered underutilized species in identical clades that can be studied further to identify nutritional value as well as cultivation practices.

Research of the phylogenetics of edible plants like our study supports arguments for the conservation of incipiently cultivated species, varietal forms and wild types of already domesticated crops (Molina-Venegas 2021; Molina-Venegas *et al.* 2021). Furthermore, the estimation of PD and identification of patterns of ethnobotanical convergence act to promote new research in ethnobiology (Gartnaje *et al.* 2017; Gaoue *et al.* 2021*a*, *b*).

Our results corroborate the importance of wild plants eaten raw or cooked in the Province of the Gulf of Mexico, demonstrating that these are an essential component of the diets of the inhabitants, providing health and economic benefits to local communities and family farmers who are engaged in their production, as has been shown in several regions around the world (Bacchetta *et al*. 2016; Gaoue *et al.* 2021*a*, *b*). Phylogenetic diversity and patterns of convergence of the type of use in the edible plants associate evolutionary history with basic human wellbeing to promote more concrete examples of the services provided directly by diversity (Proches *et al.* 2008).

## Supplementary Material

plad063_suppl_Supplementary_Material

## Data Availability

The database comprising the plant species, type of use and organ utilized as food was published in: Piedra-Malagón, E.M., Sosa, V., Angulo, D.F., Díaz-Toribio, M.H., 2022. Edible native plants of the Gulf of Mexico Province. Biodiversity Data Journal 10, e80565. https://10.3897/BDJ.10.e80565.
