# Supplementary figures and images for: Wild and cultivated comestible plant species in the Gulf of Mexico: phylogenetic patterns and convergence of type of use
Source: AoB Plants. 2023 Sep 1;15(5):plad063. doi: 10.1093/aobpla/plad063 (PMC10601390; doi:10.1093/aobpla/plad063)

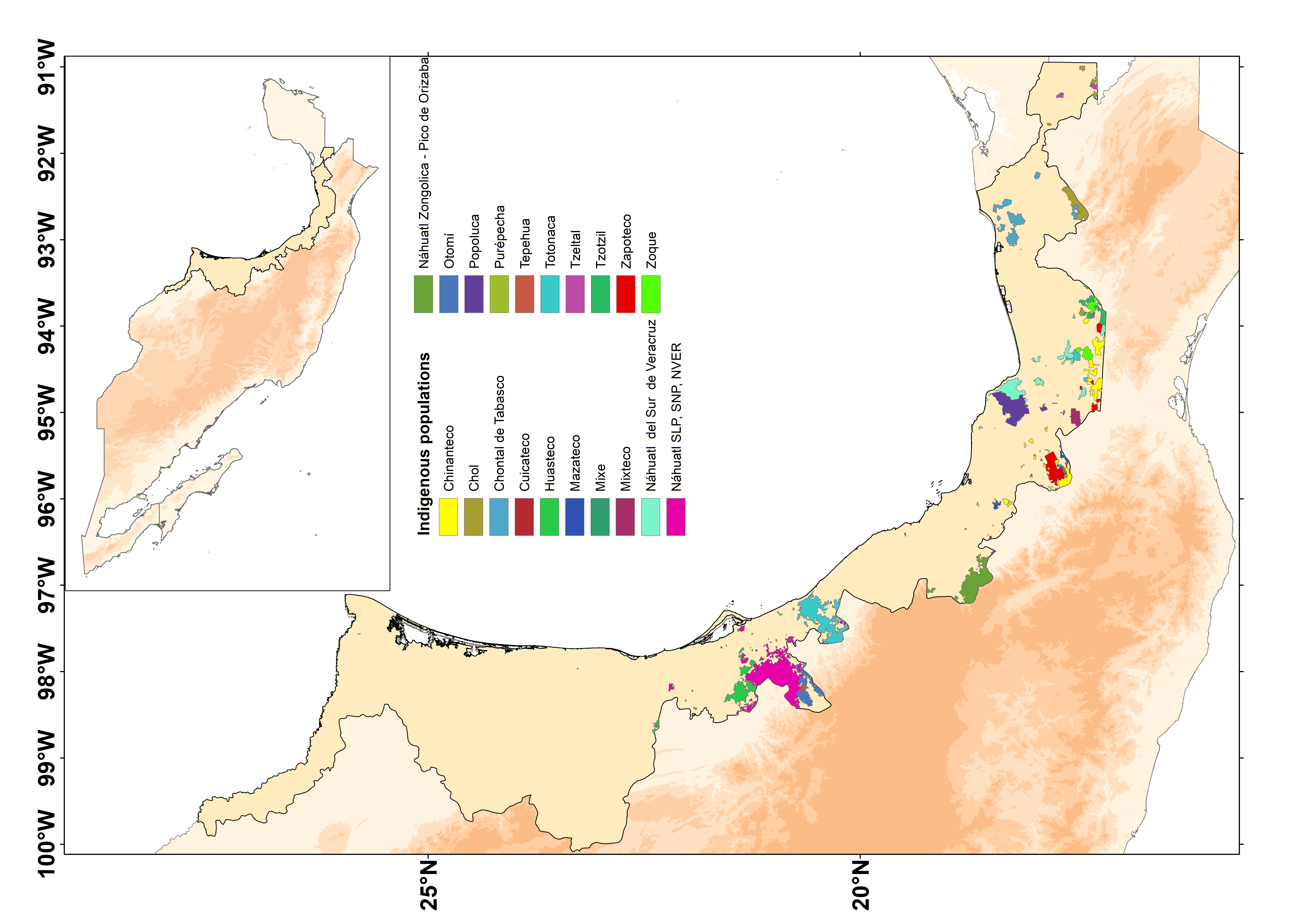

Supplement: plad063_suppl_Supplementary_Material [file plad063_suppl_supplementary_material.zip › aobplants-23045-f01-z-4c.jpg]

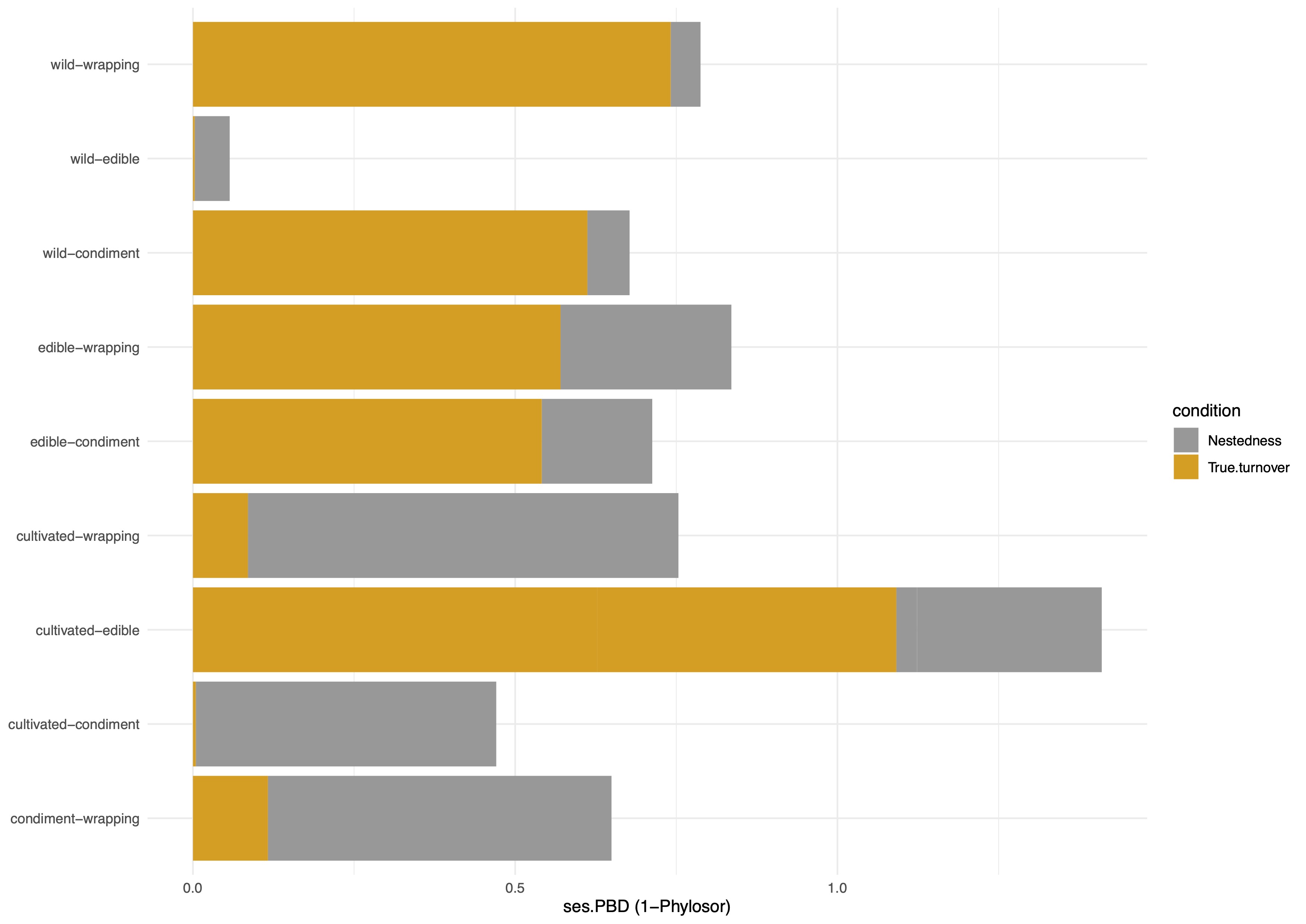

Supplement: plad063_suppl_Supplementary_Material [file plad063_suppl_supplementary_material.zip › aobplants-23045-f02-z-4c.jpg]

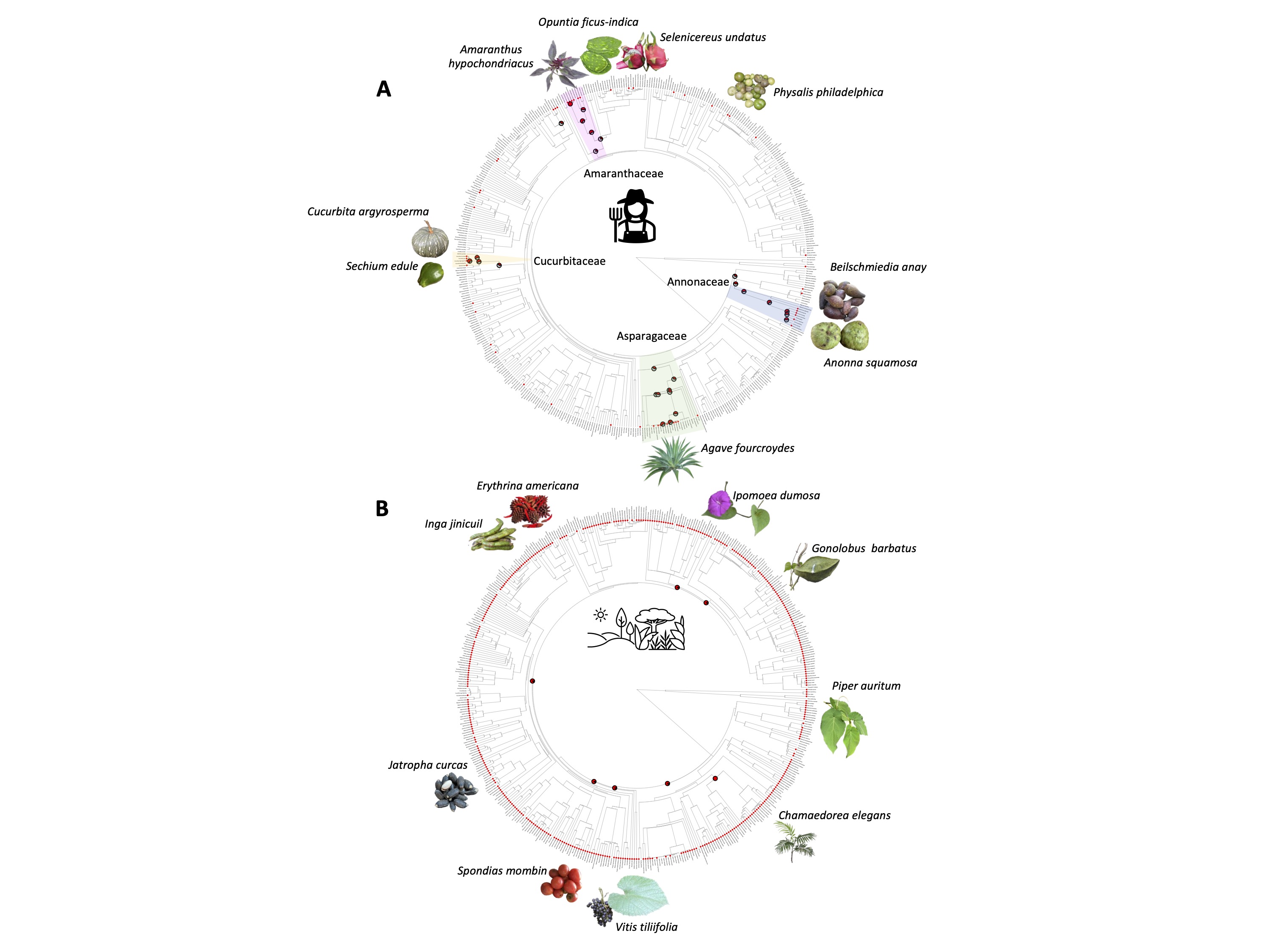

Supplement: plad063_suppl_Supplementary_Material [file plad063_suppl_supplementary_material.zip › aobplants-23045-f03-z-4c.jpg]

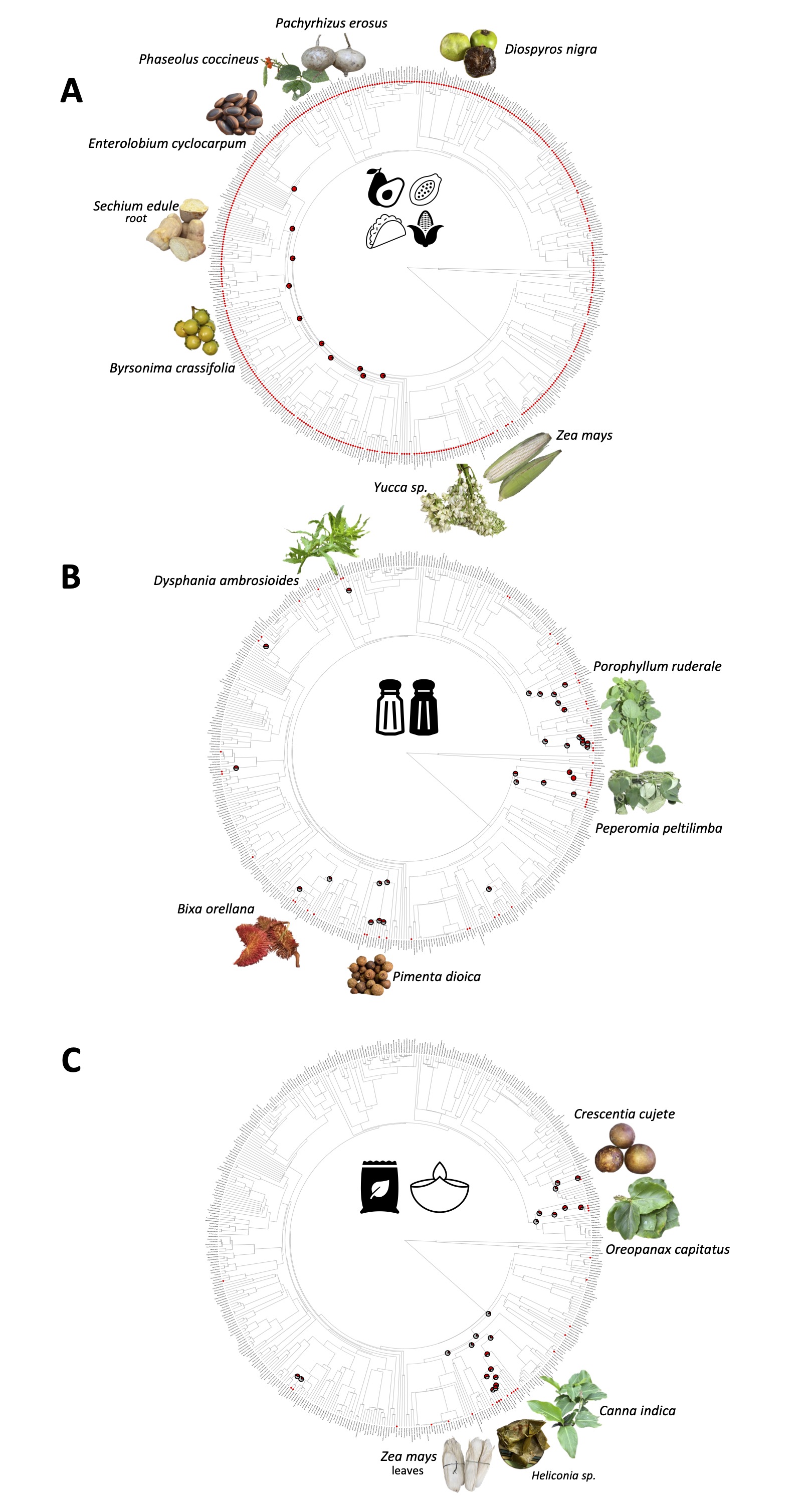

Supplement: plad063_suppl_Supplementary_Material [file plad063_suppl_supplementary_material.zip › aobplants-23045-f04-z-4c.jpg]

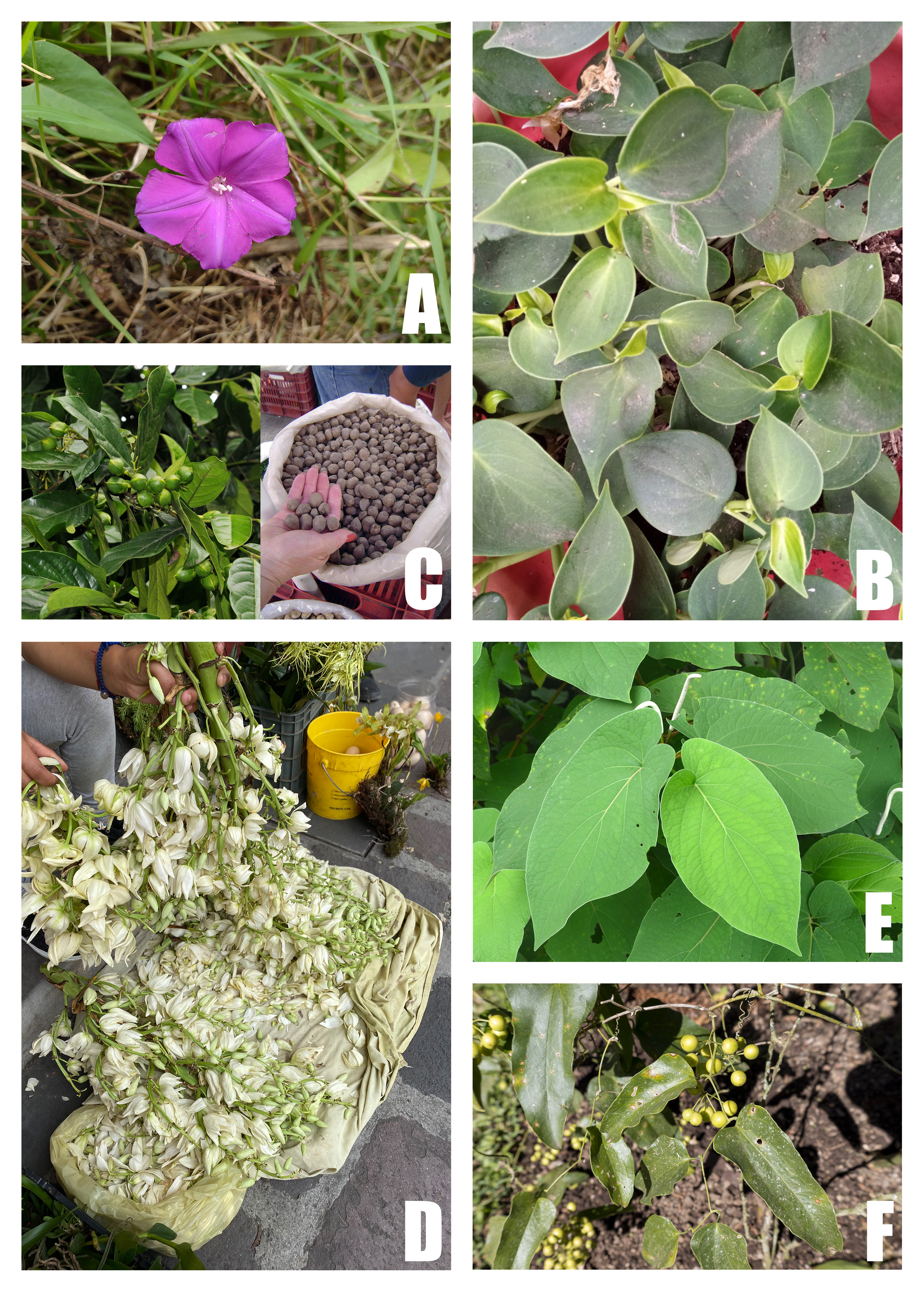

Supplement: plad063_suppl_Supplementary_Material [file plad063_suppl_supplementary_material.zip › aobplants-23045-f05-z-4c.jpg]
