# Supplementary material for: Wild and cultivated comestible plant species in the Gulf of Mexico: phylogenetic patterns and convergence of type of use
Source: AoB Plants. 2023 Sep 1;15(5):plad063. doi: 10.1093/aobpla/plad063 (PMC10601390; doi:10.1093/aobpla/plad063)
Supplement: plad063_suppl_Supplementary_Material [file aobplants-23045.pdf › aobplants-23045.pdf]

Wild and cultivated comestible plant species in the Gulf of Mexico: phylogenetic patterns and convergence of type of use

Milton H. Díaz-Toribio[1], J. Arturo de-Nova[2], Eva María Piedra-Malagón[1], Diego F Angulo[3], Victoria Sosa[4]*

[1]Jardín Botánico Francisco Javier Clavijero, Instituto de Ecología AC
[2] Instituto de Investigación de Zonas Desérticas, Universidad Autónoma de San Luis Potosí
[3]Centro de Investigación Científica de Yucatán
[4]Biología Evolutiva, Instituto de Ecología AC

*Author for correspondence: victoria.sosa@inecol.mx

Abstract

Cross-cultural research on edible plants might include ecological and evolutionary perspectives to understand processes behind species selection and management. With a database of approximately 500 comestible plants of the Province of the Gulf of Mexico in Mesoamerica, phylogenetic analyses are conducted to identify convergence and phylogenetic signal of type of use and significant clustering in the resulting phylogenetic trees. Analyses considered type of management (wild/managed vs cultivated), type of use (edible, condiment, for wrapping food) and organ utilized. Elevated phylogenetic diversity and signal is expected for wild comestible taxa, indicating that peoples are using lineages across the angiosperm tree for food, resulting in broadness in diet and use of their regional resources. Main results are: 1) condiment species were identified in groups with elevated phylogenetic signal; 2) hot nodes for lineages utilized for wrapping food were found in many monocot groups as well as in epiphytes of cloud forests with leathery leaves; 3) edible taxa were identified with the highest significant clustering restricted to certain branches in the phylogeny; 4) wild and cultivated edible plants belong to identical lineages with replacement of species, implying that same plant groups known for their comestible benefits are substituted by species distributed in the Province; 5) wild vs cultivated lineages for condiment are different. Most food species in the Province belong to four families: Fabaceae, Cactaceae, Solanaceae, and Asparagaceae. Analyses discovered underutilized wild species in identical clades to managed/cultivated taxa that can be studied further to identify cultivation practices. Results suggest that people are utilizing different lineages in the angiosperm tree available locally, for particular uses, like condiment or for wrapping food. Evidence can be used to study further undervalued edible species closely related to the most common food taxa as well as for bioprospection of their nutritional content.

Keywords: condiment plants, food plants, hot nodes, Mesoamerica, phylogenetic patterns

Introduction

Mesoamerica is a region that has been considered a center of agricultural origin where many edible plants have been domesticated. Among the most prominent are amaranth, avocado, beans, maize, chilies, papaya, pumpkin, and vanilla, and these plants continue to be managed and widely cultivated (Vavilov 1926; Harlan 1971; Khouri *et al*. 2016; Pickershill 2016; Pironon *et al*. 2020). Moreover, the peoples of Mesoamerica have been and remain engaged in diverse *in situ* agricultural practices involving wild and weed species to control the availability of useful plants, most of which are edible species (Casas *et al.* 2007; Bye 1993; Vibrans 2016). Perhaps the most remarkable comestible wild and weedy plants in this region are "quelites", which are edible greens closely associated to cornfields, crops and agricultural farming systems of nutritional importance (Bye 1981; Vyeyra-Odilón and Vibrans 2001; Carvalho and Barata 2016). Further noteworthy examples in Mesoamerica are several flowers and fruits gathered in natural ecosystems forming part of the people's diet such like yucca flowers and sugar apple fruit (Núñez-Colín *et al*. 2008; Sotelo *et al*. 2007; Pérez-Negrón *et al*. 2014; Mapes and Basurto 2016; Figueredo-Urbina *et al*. 2021). Moreover, the wide variety of Mesoamerican edible plants (wild, managed and cultivated) has contributed enormously to the great diversity of traditional Mexican cuisine, which is considered an intangible heritage of humanity. Therefore, due to the relevance of comestible plants in Mesoamerica we determine whether phylogenetic patterns differ in wild/managed vs cultivated species as well as in type of use as wrapping, condiment or food in a biogeographic region within Mesoamerica: the Province of the Gulf of Mexico.

Humans have access to a broad spectrum of food plants, varying to some extent as a result of cultural and plant regional diversity (Prescott-Allen and Prescott-Allen 1990). Cross-cultural research on diversity in the plant diet of humans is a field that combines anthropology, history, sociology, as well as ecological and evolutionary perspectives, in order to understand the processes behind species selection and management (Proches *et al.* 2008; Alburquerque *et al*. 2015). Furthermore, discovery of phylogenetic patterns in edible plants reveals crucial ecological practices and illustrates the breadth of human diets (Proches *et al.* 2008). Phylogenetic analyses comprising edible species can identify patterns of incidence of concurrent nodes in cladograms (Gartnaje *et al*. 2017; Molina-Venegas *et al*. 2020). Likewise, estimation of the phylogenetic diversity of edible plants within a region is useful for understanding the extent of human diets associated with ethnic groups (Van Wyk 2019; Molina-Venegas *et al.* 2020; Cordero *et al*. 2021; Pironon *et al.* 2020; León-Lobos *et al.* 2022).

Research on food plants around the world has found that different peoples prefer certain lineages, and that associations exist between clades and edible usage (Van Wik 2019, Molina-Venegas *et al.* 2020, Gaoue *et al.* 2021a, b; Cantwel-Jones et al. 2022). In the Province of the Gulf of Mexico, out of the approximately five hundred food recorded plants, most species belong to four different families: Fabaceae, Cactaceae, Solanaceae, and Asparagaceae (Piedra-Malagón *et al*. 2022). Moreover, this Province extends along the coastal plain of the Gulf of Mexico reaching altitudes up to six thousand meters above sea level (see Piedra Malagón *et al.* 2022) (Figure 1). The region with variable climate and forests from coniferous, tropical evergreen, deciduous and cloud forest to xerophilic scrubs and aquatic vegetation, represents an extensive and remarkable variable region to study the phylogenetic patterns of the edible plants where about fifteen ethnic groups inhabit this

province (Challenger and Soberón 2008; Atlas de los Pueblos Indígenas de México 2020; Piedra Malagón *et al.* 2022).

Based on the database of comestible plant species of the Province of the Gulf of Mexico, phylogenetic analyses are conducted to identify convergence and phylogenetic signal of type of use and significant clustering in the resulting phylogenetic trees. We test several hypotheses that emerge regarding this collection of data: 1) same supported plant lineages will be identified in wild vs cultivated groups of taxa although containing different species; 2) clustering of wild species utilized as condiment, as wrapping and as edible will be identified at restricted nodes of phylogenetic trees; 3) wild species utilized as condiment and for wrapping food will be identified in different lineages compared to cultivated taxa. The results will determine whether the patterns in our database are supported in phylogenetic trees and discover new valuable lineages and underutilized species. Furthermore, they are important in terms of drawing attention to underestimated comestible plants, to understand selection of food plants in the Gulf of Mexico and diversification of diets in this region, as has been demonstrated previously for other regions (Sotelo et al. 2007; Ulian *et al*. 2020; Georgiadis 2022).

2 Material and Methods

Database

Wild and managed/cultivated vascular plants, classed as food (edible, condiment, or wrapping), considering plant organs utilized (roots/bulbs/rhizomes, stems, bark, wood, latex/resin, leaves, flowers, fruits, seeds) were considered (Piedra-Malagón *et al.* 2022). Information was based on fieldwork conducted as part of our project, the relevant published literature, and data included with specimens deposited in the herbaria of this Province (Piedra-Malagón et al. 2022). The database comprised 473 species of vascular plants distributed in the Province of the Gulf of Mexico and was utilized to carry out analyses. The nomenclature was corrected from the initial number of the published database (487), based on World Flora Online (www.worldfloraonline.org) (Piedra-Malagón *et al.* 2022).

Phylogeny reconstruction

A recently published mega-phylogeny known as "GBOTB" was the basis on which a phylogeny was generated for the edible species recorded in the Province of the Gulf of Mexico. GBOTB had been constructed with 79,881 taxa in GenBank and using the backbone provided by Open Tree of Life (Smith and Brown 2018). This constitutes the most comprehensive and up-to-date time-calibrated species-level mega-phylogeny for seed plants. To build the phylogeny for the edible species, Phylomatic and BLADJ approaches (Webb *et al.* 2008) implemented in the V.PhyloMaker (scenario 3) were utilized (Jin and Qian 2019). V.PhyloMaker places any missing species at the basal node within a given genus, and any missing genera at the basal node within their respective families. We pruned the phylogeny in order to retain only the species present in our database.

Phylogenetic signal

To test phylogenetic signal, type of management, type of use and organ utilized were coded for every species and analyses conducted based on the phylogenetic tree already constructed. Separate analyses were conducted for every type of use and management and for all uses. The method developed by Fritz and Purvis (2010) was selected to estimate phylogenetic dispersion (D) for discrete traits, with the R package 'caper' (Orme *et al.*

2018). We performed 1000 permutations based on random patterns and these were compared to the observed phylogenetic pattern distributions for each food category and plant organ utilized in order to evaluate whether this value differed significantly from that expected with no (random) phylogenetic structure. A decrease in the values of D from 1 increases phylogenetic clumping in the binary trait; therefore, a D statistic value of 0 indicates that the trait is phylogenetically conserved (phylogenetic signal), as would be expected under a Brownian model of trait evolution, while a value of 1 suggests a random mode of evolution (no phylogenetic signal) (Fritz and Purvis 2010). If the observed D value is found between 0 and 1, but with significant departure from 1 (random), this means the trait is non-random along the phylogeny. Otherwise, the trait is considered random. The phylogenetic signal in the studied characters was graphically illustrated using the "contmap" function of the phytools package (Revell 2012) of R. All analyses were performed using R version 3.6.0 (R Core Team 2019).

Phylogenetic diversity and clustering

Phylogenetic diversity *sensu stricto* (PD), the mean pairwise phylogenetic distance (MPD), and the mean pairwise distance to the nearest taxon (MNTD), were estimated for every type of use (edible, condiment, wrapping) and for wild/cultivated species. PD and MNTD are terminal metrics reflecting phylogenetic structure that is dominant near the tip of the tree, while MPD considers basal branches of the phylogeny (Webb et al. 2008; Mazel et al., 2016). The standardized effect sizes of these metrics were determined to explore phylogenetic structure of type of use guilds since they test significant phylogenetic clustering at the full phylogeny, as well at the basal and terminal branches (Webb *et al.* 2002; Mazel et al., 2016). Moreover, they quantify the relative excess (overdispersion) or deficit (clustering) in phylogenetic diversity for a given set of species for each type of use relative to species pool in the full phylogeny. A negative standardized metric reflects a relative clustering of species while a positive standardized metric reflects a relative overdispersion of species (Mazel *et al*. 2016). Analyses were performed in Picante (Kembel et al. 2010) for R v.4.0.5 (R Core Development Team 2021). Significant phylogenetic clustering was detected when p values of the standardized metrics were below 0.05 and significant overdispersion when p values were above 0.95, equivalent to a standard effect size >1.96 or <-1.96 (Mazel et al., 2016).

To assess the degree of phylogenetic overlap between clusters of wild vs cultivated groups and groups according to type of use we explored phylogenetic beta diversity (PBD) using the PhyloSor index (Bryant *et al.* 2008) as a distance metric (see Molina-Venegas *et al*. 2020). PBD is defined as 1 – PhyloSor index and can be decomposed into two additive components, namely 'true' phylogenetic turnover (hereafter 'turnover') and nestedness, which represent different aspects of beta diversity (Leprieur *et al.* 2012; Molina-Venegas *et al*. 2020). We evaluated if the observed turnover component of PBD was higher (ses.PBD>1.96) than expected for a given comparison by computing SES scores for the phylogenetic turnover (ses.$pß_{sim}$) measured as Simpson derived pair-wise phylogenetic dissimilarity. The nestedness-resultant phylogenetic dissimilarity was measured as the nestedness-fraction of Sorensen (ses.$pß_{sne}$ derived pair-wise phylogenetic dissimilarity, and for phylogenetic beta diversity (ses.$pß_{sor}$) it was measured as Sorensen derived pair-wise phylogenetic dissimilarity (Molina-Venegas et al., 2020). Higher than expected values would indicate that the replacement involves deeper nodes of the phylogenetic tree (i.e., low phylogenetic overlap). For these analyses, we used the code ses.phylo.beta.pair

provided by Molina-Venegas et al., (2020) and the package betapart (Baselga 2010; Baselga *et al.* 2018) for R v.4.0.5 (R Core Development Team 2021).

Hot nodes and patterns of convergence of type of use

Plant groups with a significantly high number of edible species were identified through the search for "hot nodes" in the phylogenetic tree. Analyses were conducted for: 1) all edible plants in the Province of the Gulf of Mexico, 2) all edible plants considering each food use type (edible, condiment, wrapping); and 3) wild/cultivated species. For each use type, the number of comestible species descending from each node of the phylogeny was recorded and compared to a null distribution of values generated by shuffling trait values across the tips of the phylogeny 999 times (Saslis-Lagoudakis *et al.* 2011; Molina-Venegas *et al.* 2020). For a nominal alpha of 5%, the richness of edible plants in clade I will be higher than expected for the given null model if the corresponding SES score is > 1.96. We only evaluated those clades that included ten species or more, since previous studies have documented unacceptable rates of statistical errors for smaller lineages (Parra *et al.* 2010). The computer code to conduct the hot node analysis was provided by Molina-Venegas *et al.* (2020) and the analysis was performed in R v.4.0.5 (R Core Development Team 2021).

Results

Phylogenetic signal

Results of the analyses to determine phylogenetic signal using the D statistics test are presented in Table 1. These were conducted for all comestible species in the Province of the Gulf of Mexico (cultivated/wild-managed species), as well as separately for cultivated and managed/wild species, taking into account the type of use (edible, condiment, wrapping) and particular organ utilized (root/rhizome/bulb, stem, bark wood, leaf, flowers, fruits, seeds, resin/latex). Evidence of phylogenetic signal was determined in all edible plants, considering food use type (Table1): A) edible (D = 0.37, P_{rand} = <0.057), B) condiment (D = 0.47, P_{rand} = <0.004) and C) wrapping (D = 0.25, P_{rand} = <0.17). The only one of these categories that presented phylogenetic significance was condiment. Evidence of significant phylogenetic signal was detected in three plant organs utilized (root/rhizome/bulb, leaf and seeds). Phylogenetic signal in roots or bulbs (D = 0.43, P_{rand} = <0.031, Table 1) was found in families such as Smilacaceae (6 spp.), Euphorbiaceae (4 spp.), and Araceae (3 spp.). Phylogenetic signal in stems (D = 0.56, P_{rand} = <0.001, Table 1) was related to clades formed by families such as Cactaceae (8 spp.), Piperaceae (6 spp.), and Cucurbitaceae (4 spp.). Phylogenetic signal for leaves (D = 0.37, P_{rand} = <0.002, Table 1) was found in families such as Asparagaceae (11 spp.), Amaranthaceae, Leguminosae, and Piperaceae (7 spp.), Asteraceae and Solanaceae (6 spp.). Phylogenetic signal in flowers (D = 0.37, P_{rand} = <0.002, Table 1) was found in species of families such as Asparagaceae (21 spp.), Leguminosae (20 spp.), Arecaceae (9 spp.), and Cucurbitaceae (6 spp.). Phylogenetic signal in fruits (D = 0.02, P_{rand} = <0.593, Table 1) was found in species of families such as Leguminosae (32 spp.), Solanaceae (30 spp.), Cactaceae (24 spp.), Sapotaceae (14 spp.), Annonaceae (12 spp.), and Araceae, Myrtaceae, and Rosaceae (11 spp.). Phylogenetic signal in seeds (D = 0.39, P_{rand} = <0.011, Table 1) was found in species of families such as Leguminosae (13 spp.), Malvaceae (9 spp.), Cucurbitaceae (6 spp.), and Amaranthaceae, Arecaceae and Juglandaceae, with four species per family. Phylogenetic signal in resin or latex (D = 0.31, P_{rand} = <0.37, Table 1) was found in species of the families Sapotaceae (3 spp.) and Leguminosae (10 spp.). In the case of bark (D = -0.06, P_{rand} = 0.57, Table 1) and

wood (*D* = -0.47, P_{rand} = 0.63, Table 1), the phylogenetic signal was not significant. For analyses carried out considering only wild and exclusively cultivated taxa, the results for cultivated species showed that only the condiment food type presented a strong phylogenetic signal (D= 0.87 $P_{rand=}$ 0.015, Table 1), with the use of stems found to be significant (*D*=1.04, P_{rand} = 0.002, Table 1). For wild species, the uses of stems (D= 0.56, P_{rand} = 0.002, Table 1), leaves D=0.42, P_{rand} = 0.002) and seeds (D=0.41, P_{rand} = 0.011) were significant.

Phylogenetic diversity and phylogenetic clustering

The edible type of use was identified with the highest PD; in contrast condiment had the highest MPD, while wrapping the highest MNTD (Table 2). Standardized, ses.PD values indicate phylogenetic clustering for type of food (wrapping, condiment, edible) with negative and significative values ($p<0.05$). Basal phylogenetic clustering was detected only in wrapping (ses.MPD p values $p<0.05$), and terminal phylogenetic clustering in condiment and wrapping (ses.MNTD p values <0.05) (Table 2). Phylogenetic metrics in wild taxa were higher in comparison with cultivated species, showing significant overdispersion in the entire phylogeny (positive values of PD; $p>0.95$). Phylogenetic dissimilarities among type of use, estimated by PBD analyses were higher than expected (>0.64) with a true turnover component in edible vs condiment (0.54) and edible vs wrapping (0.57) comparisons (Table 3). Scores for ses.pßsim and ses.pßsor were >1.96 for edible vs condiment, edible vs wrapping, and condiment vs wrapping, indicating that the replacement of species involves deeper nodes of the phylogenetic tree with significant low overlap between these comparisons. In contrast, the nestedness component was higher (0.82) in condiment vs wrapping indicating high phylogenetic overlap between them (Table 3). Comparisons between type of use vs wild or cultivated indicate a higher true turnover component in edible vs cultivated (0.62), condiment vs wild (0.61), and wrapping vs wild (0.74) Figure 2 displays these results, indicating whether significant nestedness vs true turnover occurred in the phylogenetic trees. Nestedness and true turnover for the comparison between wild vs cultivated taxa found that species are nested in the same lineages although they were different taxa (Figure 2). For species utilized for wrapping food and as condiment they were not nested suggesting that they belong to different lineages (Figure 2).

Hot nodes and convergence of type of use

The results of hot nodes revealed several important hot nodes grouping some lineages with a clear pattern of convergence in use (Fig. 3A). These include the node of Magnoliids, which comprises lineages such as Annonaceae (*Annona*: 5 spp., *Mosannona*) and several Piperaceae (*Piper* spp., *Peperomia*) and Lauraceae (*Persea* spp., *Beilschmiedia* sp.). A second node was identified in the order Caryophyllales, which includes species of Amaranthaceae (*Amaranthus* spp., *Atriplex* sp., *Chenopodium* sp., *Dysphania* sp.) and Cactaceae (*Myrtillocactus* sp., *Opuntia* spp., *Selenicereus* sp., *Stenocereus* sp.). An important node included taxa in Fabaceae. In addition, a hot node was determined in a clade in Cucurbitaceae comprising species of *Cucurbita* and *Sechium*, as well as a clade in Asparagales comprising *Agave* spp. and *Yucca* spp. For the analyses that exclusively considered wild/cultivated species, seven “hot nodes” were evidenced; however, the most significant of these was found in the Monocots (Fig. 3B). This node comprised species utilized for food wrapping in the order Zingiberales: Cannaceae, Heliconiaceae and

Marantaceae, as well as in order Arecales, in which several species of *Chamaedorea* present edible inflorescences. Regarding use type, edible species were scattered throughout the phylogenetic tree with hot nodes including many taxa in several lineages (Fig. 4A). For the phylogenetic tree presenting species used as condiments (Fig. 4B), two important hot nodes were determined. The first featured species in Piperaceae (*Piper* spp., *Peperomia* spp.), with a sister group comprising Lauraceae (*Licaria* sp., *Persea* spp.), and the second included several species in Asteraceae (*Tagetes* spp., *Dahlia* sp., *Porophyllum* sp.). There were also some small hot nodes; one of which included *Prosopis* spp. in the Fabaceae and *Dysphania* spp. in the Amaranthaceae. The phylogenetic tree comprising the species utilized for wrapping food presented two main hot nodes (Fig. 3C); one grouped species in *Oreopanax* in the Araliaceae and the other included lineages in the Monocots. *Zea mays, Calathea* sp., *Renealmia* sp., *Stromanthe* sp., *Heliconia* spp., *Canna* sp.) were also identified in these lineages.

Discussion

Phylogenetic analyses and hot nodes

Species utilized as condiment were identified in lineages with elevated support and in significant hot nodes. Two groups stand out: Piperaceae and Asteraceae. The flavor of Piperaceae species is coriander-like and they have been used as condiments in many dishes since pre-Hispanic times (Picó and Nuez 2000; Lascurain-Rangel *et al.* 2022). Species are epiphytes common to cloud forests of the central area of the Province of the Gulf of Mexico (Vergara-Rodríguez *et al.* 2017). In addition, a third hot node lies in the Lamiaceae, in which several species of *Salvia* formed a group. Use of these taxa for providing a sage-like flavor to food has been recorded since pre-Hispanic times (Picó and Nuez 2000).

Furthermore, it is worth mentioning that hot nodes for lineages utilized for wrapping food, were found in many monocot groups. Broad leaved species in *Heliconia*, *Canna* and *Maranta* are utilized for wrapping tamales (Lascurain *et al*. 2017). Tamales are made of maize dough (sweet, salty or sour) with raisins or vegetables or meat with chili, wrapped with these leaves (Lascurain *et al*. 2017). In addition, another lineage for covering tamales with ample and leathery leaves in *Oreopanax* spp. of the Araliaceae was identified in a hot node (Lascurain *et al.* 2017). All these species possess similar anatomical characteristics featuring leathery leaves, highlighting the relevance of phenotypical convergence in human selection.

Species in lineages in cultivated and weedy/wild plants in which the stems and leaves are edible greens, the "quelites", widely consumed in Mexico, were identified with elevated phylogenetic signal as well (Bye 1981; Linares *et al.* 2017). In the order Caryophyllales, the most important sub-node included quelites in genera such as *Amaranthus*. Amaranth is known as a pseudocereal and has been widely utilized in central Mexico since pre-Hispanic times (Marx 1977); however, its leaves, stems and inflorescences are eaten as quelites prepared in many dishes (Linares *et al.* 2017). Fruit species in the cucurbits *Sechium* and *Cucurbita* spp. and eight species of cherimolas or sugar apples, *Annona* spp. were identified in hot nodes in different lineages. These taxa have been amply recorded for several regions in Mesoamerica (Basurto-Peña *et al.* 2003; Escobedo-López *et al*. 2019).

Most of the hot nodes identified in the phylogeny coincided with lineages in the most diverse families identified in the Province of the Gulf of Mexico as comestible:

Fabaceae, Cactaceae, and Asparagaceae (Piedra-Malagón *et al.* 2022). Furthermore, approximately 12% of the species recorded in this Province are cultivated while approximately 88% are collected in the wild or under incipient management (Piedra-Malagón *et al.* 2022).

Phylogenetic diversity and clustering

Regarding type of use, species in lineages classed as edible were identified with the highest phylogenetic diversity (PD), they represent the majority (97%) of species and belong to diverse lineages thus reflecting evolutionary heterogeneity. It has been documented that the diversity of edible plants is related to biocultural processes as well as ecological variation (Murphy and Fuller 2016; Carvalho and Barata 2016). In the Province of the Gulf of Mexico fifteen ethnic groups and variation in vegetation exists, therefore it was expected that many plant groups are eaten or utilized as condiment or for wrapping food.

Wild comestible taxa were identified with the highest significant clustering (MPD) restricted to certain branches in the phylogeny. Same lineages were identified again like in previous analyses. Species in the order Caryophyllales and species in *Annona* were grouped in clades of terminal branches of the phylogenetic trees. In Caryophyllales, different lineages in Cactaceae known for their edible fruits, such as pitahayas (*Selenicereus* spp.) and prickly pears (*Opuntia* spp.) (Ramírez-Rodríguez *et al.* 2020), or in Cucurbitaceae, with many edible fruits such as chayote (*Sechium edule*) or pumpkin (*Cucurbita* spp.) (Segura *et al*. 2018), were clustered in nodes with elevated MNTDs values. It is worth mentioning that species utilized for wrapping food and for condiment displayed high MNTDs as well, indicating basal or terminal clustering in cladograms. Furthermore, analyses that identify turnover and nestedness of clustering confirm these results. For wild species utilized as wrapping and for condiment, turnover is evident, meaning that lineages between species with this type of use are not shared between wild and cultivated clusters.

Phylogenetic dissimilarities quantified by PBD (phylogenetic beta diversity) displayed significantly deep phylogenetic true turnover (i.e., low phylogenetic overlap) between edible lineages vs condiment lineages and for groups for wrapping food. Interestingly, true turnover in comparisons with managed plants was only detected in the edible category. We consider this pattern, in addition to phylogenetic clustering, as evidence of similar uses for plants included in certain nodes of the phylogeny.

Implications of phylogenetic results

Phylogenetic analyses carried out with the approximately 500 comestible plant species in this study, helped to uncover ecological processes and selection by peoples in the Province of the Gulf of Mexico. Elevated phylogenetic diversity determined in analyses including all species (wild/managed + cultivated) indicates that peoples are using lineages across the angiosperm tree for food, suggesting ample breadth in the people's diet (Proches *et al*. 2008). Furthermore, species utilized for food vary according to culture, time and region (Prescott-Allen and Prescott-Allen 1990). Our study identified several lineages scattered in the angiosperm tree used for food that are distinctive of certain ecosystems in the Province of the Gulf of Mexico such as cloud forests, characterized by abundant epiphytes (Hietz and Hietz-Seifert 1995). Species in epiphytic genera such like *Peperomia* are utilized as condiment (Lascurain-Rangel *et al.* 2022), or like *Oreopanax*, a distant group in the phylogeny, which leaves are collected in the field for wrapping tamales (Lascurain *et al.* 2017). These results indicate that people are utilizing different lineages in the angiosperm

tree available locally, for particular uses, like condiment or for wrapping food; moreover, phylogenetic metrics support these groupings depicted from the database. Convergence of type of use in lineages suggest that taxa possess either similar nutritional or flavoring compounds or morphological attributes in leaves for wrapping food.

Furthermore, true turnover and nestedness analyses for wild vs cultivated food species, determined that different species are nested in the same lineages. As mentioned, most food species in the Province of the Gulf of Mexico belong to four families such like Fabaceae, Cactaceae, Solanaceae, and Asparagaceae (Piedra-Malagón *et al.* 2022). They are well documented around the world for their comestible use (Hedrick 1972). Interestingly cultivated and wild species belong to these same lineages in this Province, however composition of groups varies. Thus, results here discovered underutilized species in identical clades that can be studied further to identify nutritional value as well as cultivation practices.

Research of the phylogenetics of edible plants like our study supports arguments for the conservation of incipiently cultivated species, varietal forms and wild types of already domesticated crops (Molina-Venegas *et al.* 2021a, b). Furthermore, estimation of phylogenetic diversity and identification of patterns of ethnobotanical convergence act to promote new research in ethnobiology (Garnatje *et al.* 2017; Gaoue *et al*. 2021a, 2021b).

Our results corroborate the importance of wild plants eaten raw or cooked in the Province of the Gulf of Mexico, demonstrating that these are an essential component of the diets of the inhabitants, providing health and economic benefits to local communities and family farmers who are engaged in their production, as has been shown in several regions around the world (Bacchetta *et al*. 2016; Gaoue *et al.* 2021a, 2021b). Phylogenetic diversity and patterns of convergence of the type of use in the edible plants associates evolutionary history with basic human wellbeing to promote more concrete examples of the services provided directly by diversity (Proches *et al.* 2008).

Conflict of Interest Statement

None declared.

Acknowledgements

The authors are grateful to Carlos Aldair Zárate for providing images utilized in this study. Research and was funded by CONAHCYT (305103: Un Jardín Etnobiológico: Ampliando los Horizontes del Jardín Botánico Francisco Javier Clavijero en Xalapa, Veracruz). We thank Keith MacMillan for revising our English.

Data availability

The database comprising the plant species, type of use and organ utilized as food was published in: Piedra-Malagón, E.M., Sosa, V., Angulo, D.F., Díaz-Toribio, M.H., 2022. Edible native plants of the Gulf of Mexico Province. *Biodiversity Data Journal* 10, e80565. https://10.3897/BDJ.10.e80565

Literature Cited

Alburquerque, U.P., Madeiros, P.M., Casas, A., 2015. Evolutionary rthnobiology. in: Alburquerque, U.P., Madeiros, P.M., Casas, A. (Eds.), *Evolutionary Ethnobiology*. Springer, Switzerland, pp. 1‒5.

Atlas de los Pueblos Indígenas de México., 2020. Instituto Nacional de los Pueblos Indígenas/Instituto Nacional de Las Lenguas Indígenas. http//atlas.cdi.gob.mx. Accessed on: 2023-02-10.

Bacchetta, L., Visioli, F., Cappelli, G., Caruso, E., Marin, G., Nemeth, E., Bacchetta, G., Bedini, G., Wezel, A., van Asseldonk, T., van Taamsdonk, L., Mariani, F. (Eatwild Consortium). 2016. A manifesto for the valorization of wild edible plants. *Journal of Ethnopharmacology* 191, 180–187. http://10.1016/j.jep.2016.05.061

Baselga, A.m 2010. Partitioning the turnover and nestedness components of beta diversity. *Global Ecology and Biogeography* 19, 134–143. https://doi.org/10.1111/j.1466-8238.2009.00490.x

Baselga, A, Orme, D, Villeger, S, De Bortoli, J, Leprieur, F, Logez, M., 2018. betapart: partitioning beta diversity into turnover and nestedness components. R package version 1.5.0. https://CRAN.R-project.org/package=betapart.

Basurto-Peña, F., Castro-Lara, D., Martínez-Alfaro, M.A., 2003. Edible begonias from the north of Puebla, Mexico. *Economic Botany* 57, 48–53.

Bryant, JA, Lamanna, C, Morlon, H, Kerkhoff, AJ, Enquist, BJ, Green, JL., 2008. Microbes on mountainsides: contrasting elevational patterns of bacterial and plant diversity. *Proceedings of the National Academy of Sciences of the United States of America* 105, 11505–11511. https://doi.org/10.1073/pnas.0801920105.

Bye, R., 1981. Quelites- ethnoecology of edible greens- past, present and future. *Journal of Ethnobiology* 1, 109‒123.

Bye, R., 1993. The role of humans in the diversification of plants in Mexico. in: Ramamoorthy, T.P., Bye, R., Lot, A., Fa J., (Eds.), *Biological diversity in Mexico: origins and distribution*. Oxford Univ. Press, New York, pp. 707‒731.

Cantwell-Jones, A., Ball, H., Collar, D., Diazgranados, M., Douglas, R., Forest, F., Hawkins, J., Howes, M.J., Ulian, T., Vaitla, B., Pironon, S., 2022. Global plant diversity as a reservoir of micronutrients for humanity. *Nature Plants* 8, 225–232. https://10.1038/s41477-022-01100-6

Carvalho, A.M., Barata, A.M., 2016. The consumption of wild edible plants. In: Ferreira, I.C.F.R., Morales, P., Barros, L. (Eds.), *Wild plants, mushrooms and nuts: food properties and applications*. John Wiley & Sons, New York, pp. 159‒198.

Casas, A., Otero-Arnaiz, A., Pérez-Negrón, E., Valiente-Banuet, A., 2007. In situ management and domestication of plants in Mesoamerica. *Annals of Botany* 100, 1101‒1115. https://:10.1093/aob/mcm126

Challenger, A, Soberón, J., 2008. Los ecosistemas terrestres. in: Sarukhán J. (Ed.). *Capital Natural de México.* Vol 1: Conocimiento actual de la Biodiversidad. CONABIO, México D. F. pp. 87‒108.

Cordero, S., Gálvez, F., Carvallo, G.O., 2021. Biodiversity-productivity relationship in urban vascular flora: a comparison between wild edible and non-edible plants. *Botanical Sciences* 100, 107‒119. DOI:10.17129/botsci.2892

Escobedo-López, D., Campos-Rojas, E., Rodríguez, J.R., Alia-Tejacal, I., Núñez-Colín, C.A., 2019. Priority areas to collect germplasm of *Annona* (Annonaceae) in Mexico based on diversity and species richness indices. *Genetic Resources and Crop Evolution* 66, 401–413. https://10.1007/S10722-018-0718-2.

Figueredo-Urbina, C.J., Alvarez-Ríos, G., Cortés Zárraga, L., 2021. Edible flowers commercialized in local markets of Pachuca de Soto, Hidalgo, Mexico. *Botanical Sciences* 100, 12−138. https://10.17129/botsci.2831

Fritz, S.A., Purvis, A., 2010. Selectivity in mammalian extinction risk and threat types a new measure of phylogenetic signal strength in binary data. *Conservation Biology* 24, 1042−1051. https://10.1111/j.1523-1739.2010.01455.x

Gaoue, O.G., Moutouama, J.K., Coe, M.A., Bond, M.O., Green, E., Sero, N.B., Bezeng, B.S., Yessoufou, K., 2021b. Methodological advances for hypothesis driven ethnobiology. *Biological Reviews* 96, 2281–2303. https://10.1111/brv.12752

Gaoue, O.G., Yessoufu, K., Mankga, L., Vodohue, F., 2021b. Phylogeny reveals non-random medicinal organ selection by local people in Benin. *Plants People Planet* 3, 710–720. https://10.1002/ppp3.10202

Gartnaje, T., Peñuelas, J., Vallès, J., 2017. Ethnobotany, phylogeny, and 'omics' for human health and food security. *Trends in Plant Science* 22, 187−191. https://10.1016/j. tplants.01.001

Georgiadis, P., 2022. Ethnobotanical knowledge against the combined biodiversity, poverty and climate crisis. *Plants People Planet* 4, 382–391. https://10.1002/ppp3.10259

Harlan, J.R., 1971. Agricultural origins: centers and non centers. *Science* 174, 468–474. https://10.1126/science.174.4008.468

Hawkins, J. and Teixidor-Toneau I., 2017. Defining 'ethnobotanical convergence'. *Trends in Plant Science* 22, 639-640.

Hedrick, U.P., 1972. Sturtevant's edible plants of the world. Dover Publications, New York. 300 pp.

Hietz, P., Hietz-Seifert, U., 1995. Structure and ecology of epiphyte communities of a cloud forest in central Veracruz, Mexico. *Journal of Vegetation Science* 6, 719–728 https://doi.org/10.2307/3236443

Jin, Y., Qian, H., 2019. V.PhyloMaker: an R package that can generate very large phylogenies for vascular plants. *Ecography* 42, 1353−1359. https://10.1111/ecog.04434

Kembel, S.W., Cowan, P.D., Helmus, M.R., Cornwell, W.K., Morlon, H., Ackerly, D.D., Blomberg, S.P., Webb, C.O., 2010. Picante: R tools for integrating phylogenies and ecology. *Bioinformatics* 26, 1463–1464. https://doi.org/10.1093/bioinformatics/btq166.

Khouri, C.K., Achicanoy, H.A., Bjorkman, A.D., Navarro-Racines, C., Cuarino, L., Flores-Palacios, X., Engels, J.M.M., Wiersema, J.H., Dempewolf, H., Sotelo, S., Ramírez-Villegas, J., Castañeda-Alvarez, N.P., Fowler, C., Jarvis, A., Rieseberg, L.H., Struik, P.C., 2016. Origins of food crops connect countries worldwide. *Proceedings of the Royal Society B* 283, 20160792 https://10.1098/rspb.2016.0792

Lascurain, M., López-Binqüist, C., Avendaño, S., Covarrubias, M., 2017. The plants leaves used to wrap tamales in the Mexican state of Veracruz. *Economic Botany* 71, 374–379.

Lascurain-Rangel, M., Avendaño-Reyes, S., Tan, R., Caballero, J., Cortés-Arriaga, L., Linares-Mazari, E., Bye-Boettler, R., López-Binquüist, C., Avila, A., 2022. American plants used as a condiment in Mexican cuisine. *Revista Mexicana de Biodiversidad* 93, e933949 https:// 10.22201/ib.200788706e.2022.93.3949

León-Lobos, P., Díaz-Forestier, J., Díaz, R., Celis-Diez, J.L., Diazgranados, M., Ulian, T., 2022. Patterns of traditional and modern uses of wild edible native plants of Chile: challenges and future perspectives. *Plants* 11, 744. https://10.3390/plants11060744

Leprieur, F., Albouy, C., De Bortoli, J, Cowman, P.F., Bellwood, D.R., Mouillot, D., 2012. Quantifying phylogenetic beta diversity: distinguishing between 'true' turnover of lineages and phylogenetic diversity gradients. *PloS one* 7, e42760. https://doi.org/10.1371/journal.pone.0042760.

Linares, E., Bye, R., Ortega, N., Arce, A.E., 2017. Quelites: sabores y saberes, del sureste del Estado de México. Universidad Autónoma de México, Mexico City. https:// 10.22201/ib.9786073016667e.2019

Mapes, C., Basurto, F., 2016. Biodiversity and edible plants of Mexico. in: Lira, R., Casas, A., Blancas, J., (Eds.), Ethnobotany of Mexico. Springer, New York, pp 83−131. https://doi.org/10.1007/978-1-4614-6669-7

Marx, J.L., 1977. Amaranth: a comeback for the food of the Aztecs? *Science* 198, 40. https://10.1126/SCIENCE.198.4312.40

Mazel, R., Davies, T.J., Gallien, L., Renaud, J., Groussin, M., Münkemüller, T., Thuiller, W., 2016. Influence of tree shape and evolutionary timescale on phylogenetic diversity metrics. *Ecography* 39: 913–920. https//doi.org/10.111/ecog.01694

Molina-Venegas, R., Fischer, M., Mollel, N.P., Hemp, A., 2020. Connecting plant evolutionary history and human well-being at Mt. Kilimanjaro, Tanzania. Botanical Journal of the Linnean Society 194, 397–409. https://10.1093/bottlinnean/boaa049

Molina-Venegas, R., 2021a. Conserving evolutionarily distinct species is critical to safeguard human well-being. *Scientific Reports* 11, 24187. https://10.1038/s41598-021-03616-x

Molina-Venegas, R., Rodríguez, M.A., Pardo-de-Santayana, M., Ronquillo, C., Mabberley, D.J., 2021b. Maximum levels of global phylogenetic diversity efficiently capture plant services for humankind. *Ecology and Evolution* 5, 583−588. https://10.1038/S41559-021-01414-2

Murphy, C., Fuller, D.Q., 2016. The transition to agricultural production in India. in: Schug G. R. S., Walimbe R. (Eds.) *A compilation to South Asia in the past*. John Wiley, Hoboken New Jersey

Núñez-Colín, C.A., Nieto-Angel, R., Barrientos-Priego, A.F., Sahagún-Castellanos, J., Segura, S., González-Andrés, F., 2008. Variability of three regional resources of germplasm of Tejocote (*Crataegus* spp.) from central and southern Mexico. *Genetic Resources and Crop Evolution* 55, 1159–1165. https://10.1007/s10722-008-9316-z

Orme, D., 2018. The caper package: comparative analysis of phylogenetics and evolution in R. https://www.google.com/search?client=firefox-b-d&q=R+package+caper+Orme

Parra, J.L., McGuire, J.A., Graham, C.H., 2010. Incorporating clade identity in analysis of phylogenetic community structure: an example with hummingbirds. *American Naturalist* 176, 573–587.

Pérez-Negrón, E., Dávila, P., Casas, A., 2014. Use of columnar cacti in the Tehuacán Valley, Mexico: perspectives for sustainable development of non-timber forest products. *Journal of Ethnobiology and Ethnomedicine* 10, 79. https://doi.org/10.1186/1746-4269-10-79

Pickersgill, B., 2016. Domestication of plants in Mesoamerica: an archaeological review with some ethnobotanical interpretations. in Lira, R., Casas, A., Blancas, J. (Eds.), *Ethnobotany of Mexico*. Springer, New York, pp 207–231. https://10.1007/978-1-4614-6669-7_9

Picó, B., Nuez, F., 2000. Minor crops of Mesoamerica in early sources (II). Herbs used as condiments. *Genetic Resources and Crop Evolution* 47, 541–552. https://doi.org/10.1023/A:1008732626892

Piedra-Malagón, E.M., Sosa, V., Angulo, D.F., Díaz-Toribio, M.H., 2022. Edible native plants of the Gulf of Mexico Province. *Biodiversity Data Journal* 10, e80565. https://10.3897/BDJ.10.e80565

Pironon, S., Borrell, J.S., Ondo, I., Douglas, R., Phillips, C., Khoury, C.K., Kantar, M.B., Fumia, N., Soto Gómez, M., Viurel, J., Govaerts, R., Forest, F., Antonelli, A., 2020. Toward unifying global hotspots of wild and domesticated biodiversity. *Plants* 9, 1128. https://10.3390/plants991128

Prescott-Allen, R., Prescott-Allen, C., 1990. How many plants feed the world? *Conservation Biology* 4: 365–374. https://10.1111/j.1523-1739.1990.tb00310.x

Proches, S., Wilson, J.R.U., Vamosi, J.C., Richardson, D.M., 2008. Plant diversity in the human diet: weak phylogenetic signal indicates breadth. *BioScience* 58, 151–159. https://10.1641/B580209

Ramírez-Rodríguez, Y., Martínez-Huélamo, M., Pedraza-Chaverri, Ramírez V, Martínez-Tagüeña N, Trujillo J., 2020. Ethnobotanical, nutritional, and medicinal properties of Mexican drylands Cactaceae Fruits: Recent findings and research opportunities. *Food Chemistry* 312, 126073. https://20.2026/j.foodchem.2019.126073

Revell, L.J., 2012. phytools: an R package for phylogenetic compatative geology (and other things). *Methods in Ecology and Evolution* 2, 217–223. https://soi.org/10.1111/j.2041-210X.2011.001.00169-x

Saslis-Lagoudakis, C.H., Kitgaard, B.B., Forest, F., Francis, L., Savolainen, V., Williamson, E.M., Hawkins. J.A., 2011. The use of phylogeny to interpret cross-cultural patterns in plant use and guide medicinal plant discovery: an example from *Pterocarpus* (Leguminosae). *Plos One* 6, E22275. https://10.1371/journal.pone0022275

Segura, S., Fresnedo, J., Mathuriau, C., López, J., Andrés, J., Muratalla, A., 2018. The edible fruit species in Mexico. *Genetic Resources and Crop Evolution* 65, 1767–1793. https://10.1007/s10722-018-0652-3

Smith, S.A., Brown, J.W., 2018. Constructing a broadly inclusive seed plant phylogeny. *American Journal of Botany* 105, 302–314. https://10.1002/AJB.1019

Sotelo, A., López-García, S., Basurto-Peña, F., 2007. Content of nutrient and antinutrient in edible flowers of wild plants in Mexico. *Plant Foods in Human Nutrition* 62, 133–138. https://10.1007/s11130-007-0054-9

Ulian, T., Diazgranados, M., Pironon, S., Padulosi, S., Liu, U., Davies, L., Howes, M.J.R., Borrell, J.S., Ondo, I., Pérez-Escobar, O., Sharrock, S., Ryan, P., Hunter, D., Lee, M.A., Batstwo, C., Tuczaj, T., Pieroni, A., Cámara-Leret, R., Noorani, A., Womdin, R.N., Miminjanov, H., Antonelii, A., Pritchard, H.W., Mattana, E., 2020. Unlocking plant resources to support food security and promote sustainable agriculture. *Plants People Planet* 2, 421–445. https://10.1002/PPE.10145

Van Wyk, B.E., 2019. The diversity and multiple uses of southern African legumes. *Australian Systematic Botany* 321, 519–546. https://10.1071/SB19028

Vavilov, N.I., 1926. Tzentry proiskhozhdeniya kulturnykh rastenii [The centres of origin of cultivated plants]. Works in Applied Botany and Plant Breeding 16, 1–248.

Vergara-Rodríguez, D., Mathieu, G., Samain, M.S., Armenta-Montero, S., Krömer, T., 2017. Diversity, distribution, and conservation status of *Peperomia* (Piperaceae) in the State of Veracruz. *Tropical Conservation Science* 10, 1–28. https://10.117/194008291770283

Vibrans, H., 2016. Ethnobotany of Mexican weeds. in: Lira, R., Casas, A., Blancas, J. (Eds.), *Ethnobotany of Mexico*. Springer, New York. pp. 287–317 https://10.1007/978-1-4614-6669-7_9

Vieyra-Odilón, L., Vibrans, H., 2001. Weeds as crops: the value of maize field weeds in the Valley of Toluca. *Economic Botany* 55, 426–443

Webb, C.O., Ackerly, D.D., McPeek, M.A., Donoghue, M.J., 2002. Phylogenies and community ecology. *Annual Review of Ecology and Systematics* 33: 475,505 https://doi.org/10.1146/annurev.ecolsys.33.010802.150448

Webb, C.P., Ackerly, D.D., Kemberl, S.W., 2008. Phylocom: software for the analysis of phylogenetic structure and trait evolution. *Bionformatics* 24, 2098–2100. https://doi.org./10.1093/bioinformatics/btn358

Figure Legends

Figure 1. Limits of the study area, the biogeographic Province of the Gulf of Mexico. The different ethnic groups located within the Province are indicated.

Figure 2. Graph displaying true’ turnover (blue asterisk) and nestedness (orange asterisk) additive components of PBD between of the wild vs cultivated species as well as type of use of comestible species in the Province of the Gulf of Mexico. SES scores >1.96 are indicated with an asterisk.

Figure 3. Phylogenetic trees displaying “hot nodes” of analyses based on the comestible species in the Province of the Gulf of Mexico. A. Tree illustrating studied edible plant species of the Province of the Gulf of Mexico. B. Tree displaying nodes for wild/incipient management edible plant taxa.

Figure 4. Phylogenetic trees displaying “hot nodes” for the comestible type of use (edible, condiment, wrapping) based on the comestible species in the Province of the Gulf of Mexico. A. “Hot nodes” for plant species eaten raw or cooked. B. “Hot nodes” for plant species utilized as condiments. C. “Hot nodes” for plant species utilized for food wrapping.

Figure 5. Remarkable edible plant species utilized in the Province of the Gulf of Mexico. A. *Ipomoea dumosa*, known as xonequi or xonegui, an edible green or quelite. B. Leaves of *Peperomia peltilimba*, known as cilantro del monte, utilized as a condiment. C. Edible fruits of *Oecopetalum mexicanum*, known as cachichín. D. Inflorescences of *Yucca sp.*, the flowers of which are eaten by removing the stamens and added to soups. E. *Piper auritum*, known as acuyo, is added to food as a condiment. F. *Smilax aristolochiifolia*, known as “zarzaparrilla” is the regional species utilized for producing root beer from rhizomes.

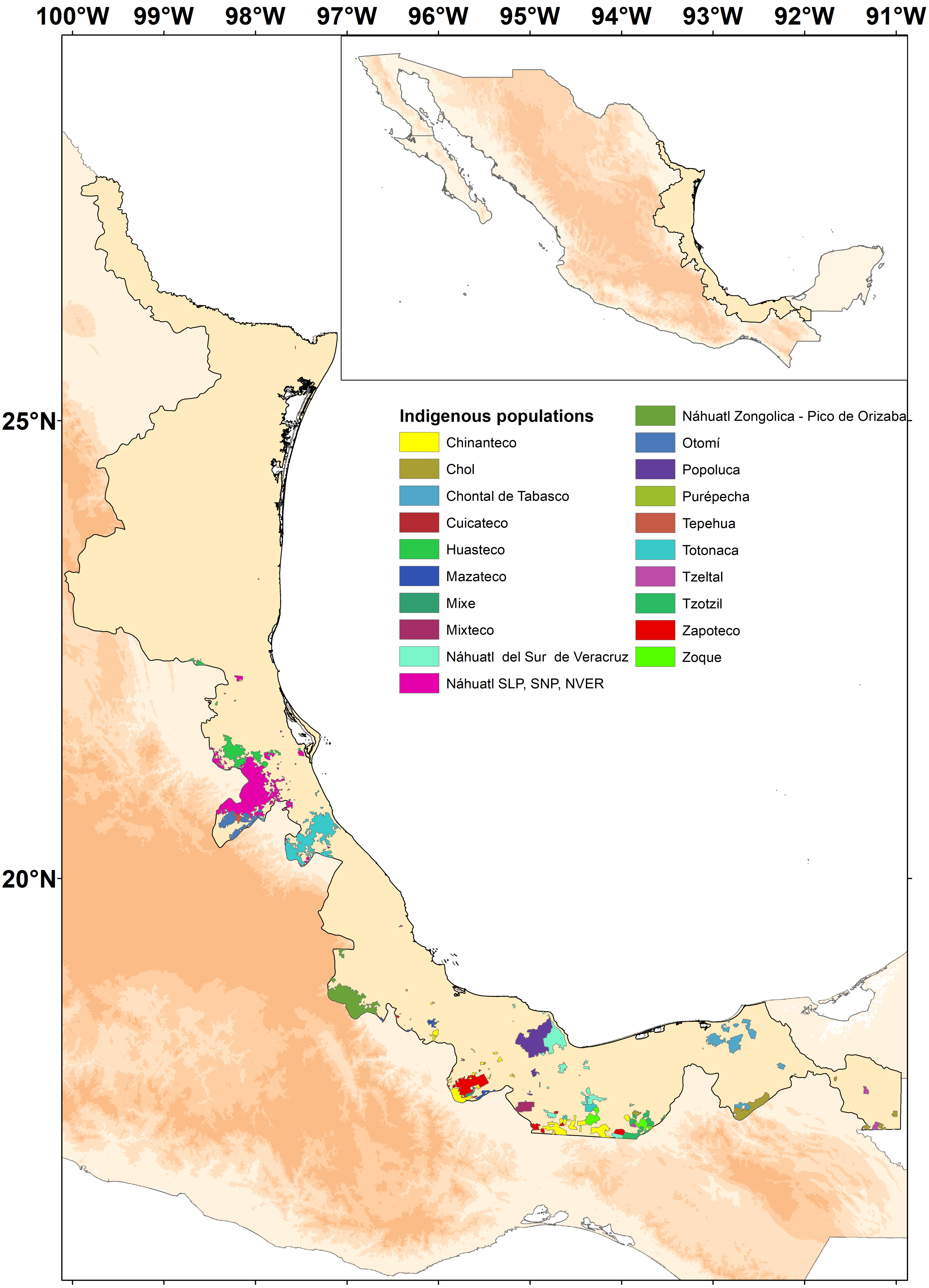

100°W
99°W
98°W
97°W
96°W
95°W
94°W
93°W
92°W
91°W
25°N
20°N
Indigenous populations
Chinanteco
Chol
Chontal de Tabasco
Cuicateco
Huasteco
Mazateco
Mixe
Mixteco
Náhuatl del Sur de Veracruz
Náhuatl SLP, SNP, NVER
Náhuatl Zongolica - Pico de Orizaba
Otomí
Popoluca
Purépecha
Tepehua
Totonaca
Tzeltal
Tzotzil
Zapoteco
Zoque

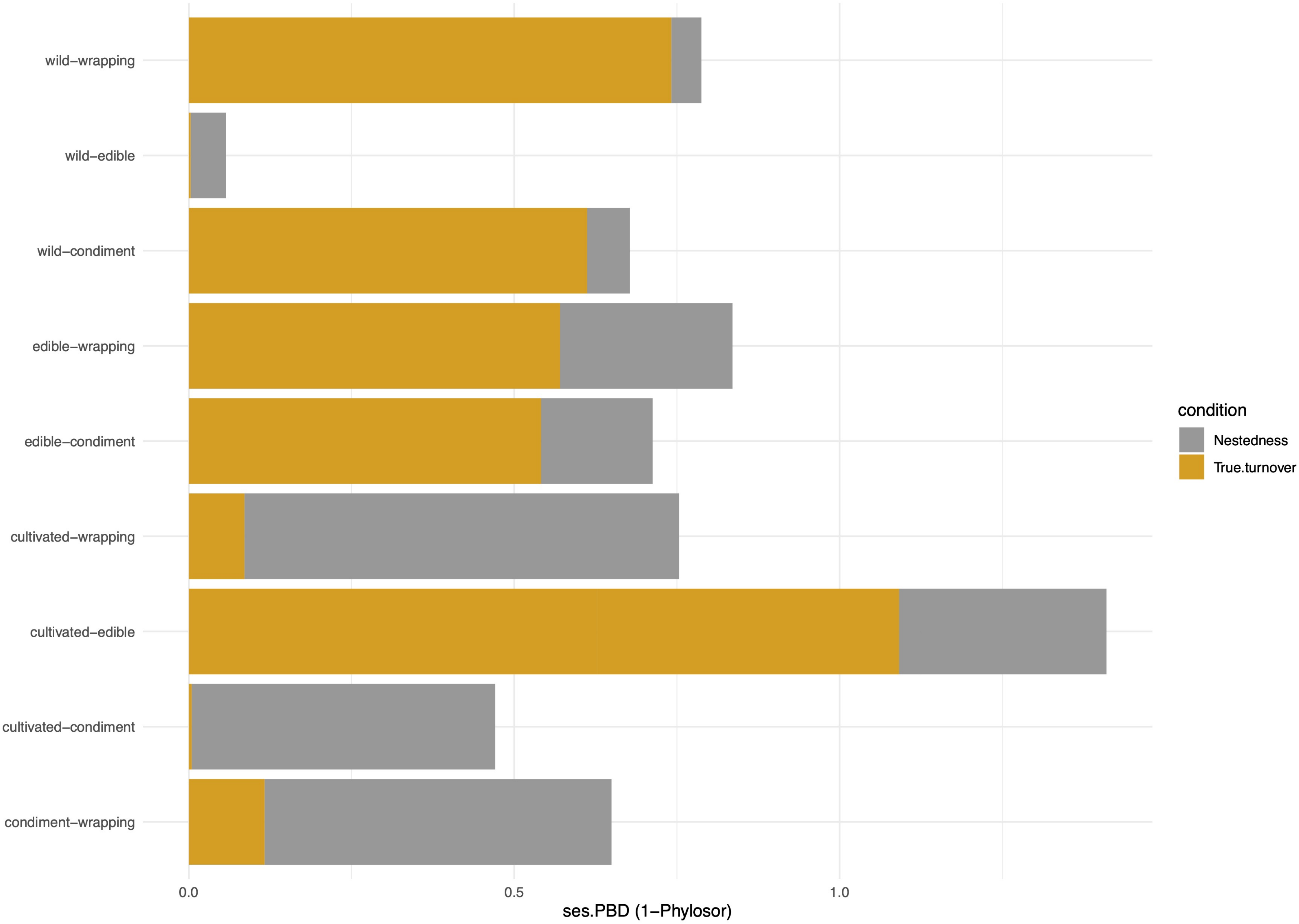
wild−wrapping
wild−edible
wild−condiment
edible−wrapping
edible−condiment
cultivated−wrapping
cultivated−edible
cultivated−condiment
condiment−wrapping
0.0
0.5
1.0
ses.PBD (1−Phylosor)
condition
Nestedness
True.turnover

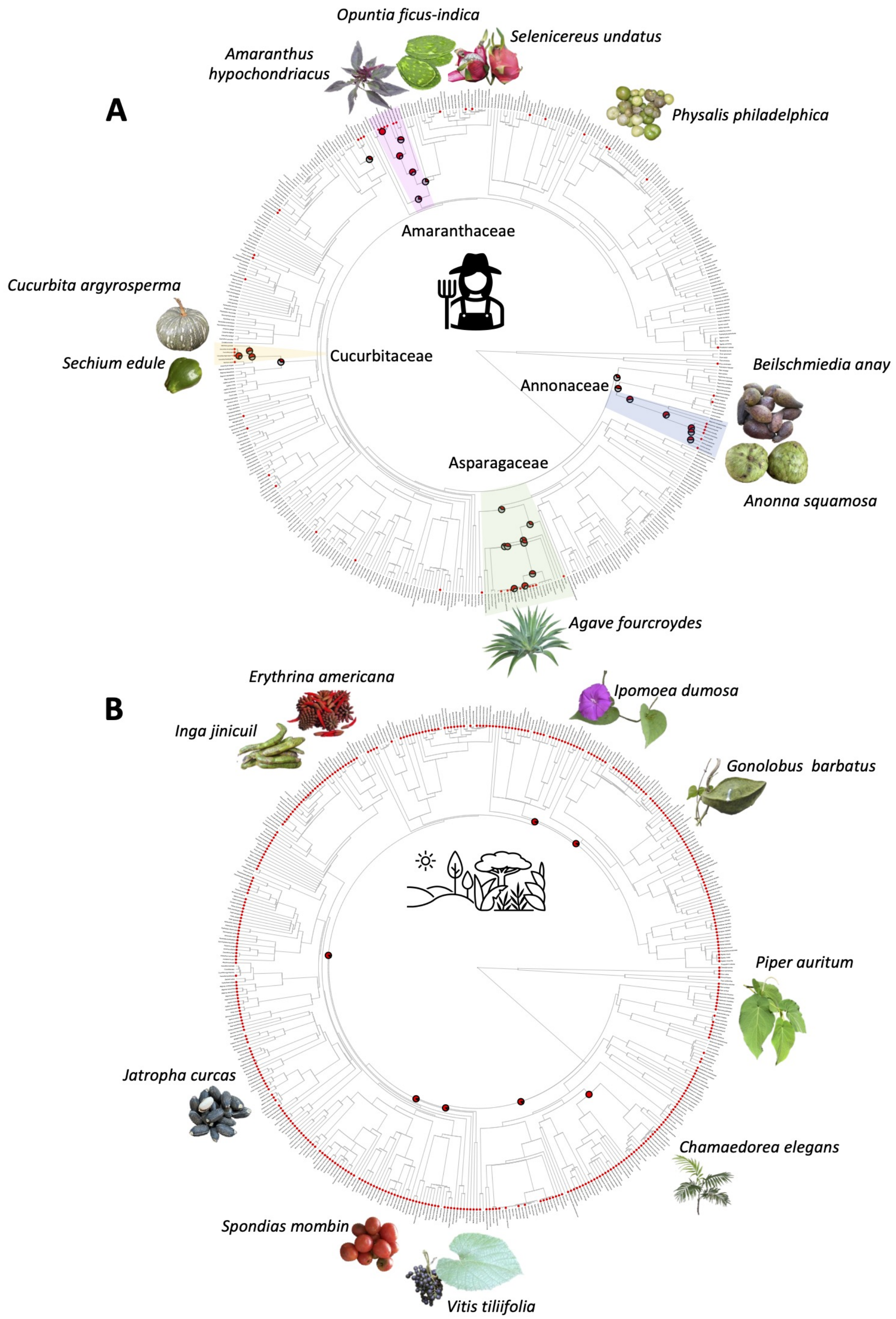
A
Amaranthus hypochondriacus
Opuntia ficus-indica
Selenicereus undatus
Physalis philadelphica
Amaranthaceae
Cucurbita argyrosperma
Sechium edule
Cucurbitaceae
Annonaceae
Beilschmiedia anay
Anonna squamosa
Asparagaceae
Agave fourcroydes
B
Erythrina americana
Inga jinicuil
Ipomoea dumosa
Gonolobus barbatus
Piper auritum
Jatropha curcas
Chamaedorea elegans
Spondias mombin
Vitis tiliifolia

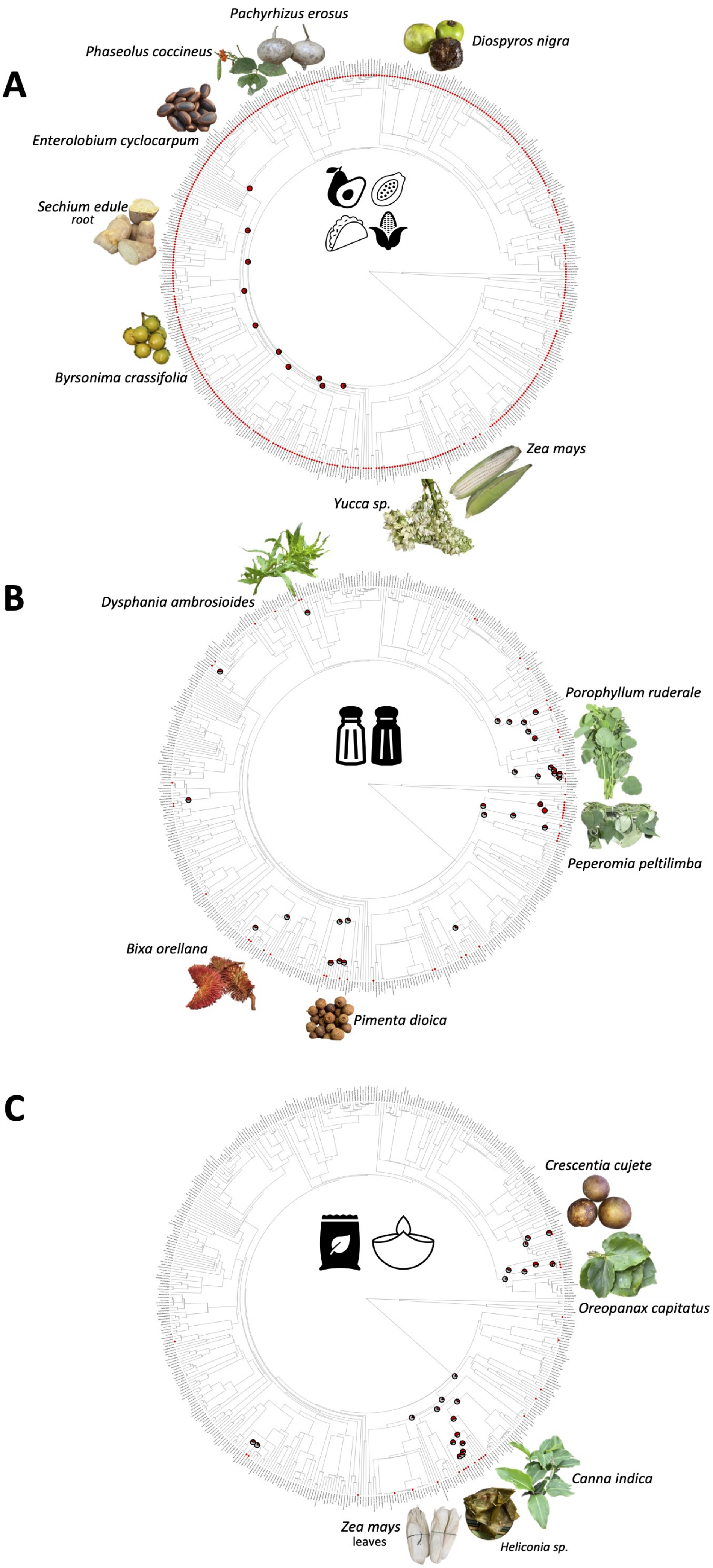

A
Pachyrhizus erosus
Diospyros nigra
Phaseolus coccineus
Enterolobium cyclocarpum
Sechium edule
root
Byrsonima crassifolia
Zea mays
Yucca sp.
B
Dysphania ambrosioides
Porophyllum ruderale
Peperomia peltilimba
Bixa orellana
Pimenta dioica
C
Crescentia cujete
Oreopanax capitatus
Canna indica
Zea mays
leaves
Heliconia sp.

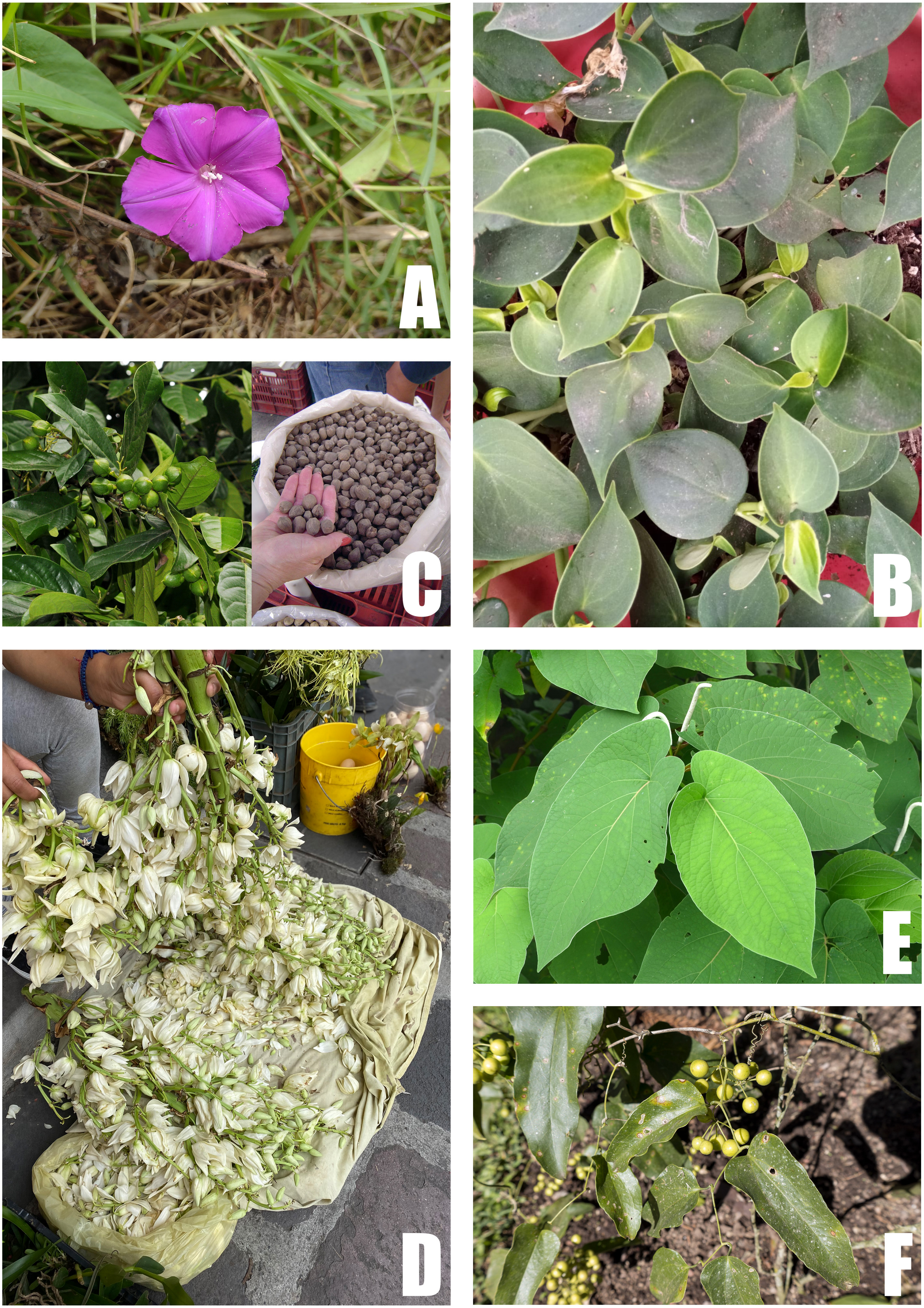
A
B
C
D
E
F

Table 1. Results of analyses utilizing the D statistics test to identify phylogenetic signal among cultivated and wild species in the edible plants of the Province of the Gulf of Mexico. Type of use and plant organ used were considered. Significant values for D and p are presented in bold.

		D	***p***
**Cultivated+Wild**	Edible	0.3748	0.057
	Condiment	**0.4794**	**0.004**
	Wrapping	0.2596	0.17
	Root/rhizome/bulb	**0.4363**	**0.031**
	Stem	**0.5627**	**0.001**
	Bark	-0.0648	0.57
	Wood	-0.4793	0.63
	Leaf	**0.3725**	**0.002**
	Flowers	0.1627	0.12
	Fruits	0.0244	0.593
	Seeds	**0.3949**	**0.011**
	Resin/latex	0.314	0.378
**Cultivated**	Edible	-0.1168	0.598
	Condiment	**0.8709**	**0.015**
	Wrapping	0.5567	0.277
	Root/rhizome/bulb	0.8858	0.215
	Stem	**1.0496**	**0.002**
	Bark	-	-
	Wood	-	-
	Leaf	0.3596	0.111
	Flowers	-0.0197	0.512
	Fruits	-0.4668	0.945
	Seeds	0.2859	0.184
	Resin/latex	-	-
**Wild**	Edible	0.3388	0.08
	Condiment	0.2661	0.098
	Wrapping	0.0702	0.428
	Root/rhizome/bulb	0.4148	0.051
	Stem	**0.5603**	**0.002**
	Bark	-0.1823	0.638
	Wood	-1.9851	0.668
	Leaf	**0.4288**	**0.002**
	Flowers	0.1277	0.222
	Fruits	-0.0035	0.514
	Seeds	**0.4137**	**0.011**
	Resin/latex	0.2194	0.429

Table 2. Species richness (SR), phylogenetic diversity (PD), mean pairwise phylogenetic distance (MPD), mean pairwise distance to the nearest taxon (MNTD), and significant values "p" estimated for the comestible plant species in the Province of the Gulf of Mexico. Standardized metrics are included as well (ses.PD, ses.MPD, ses.MNDT). Significant p values are marked with an asterisk.

Type of use	SR	PD	MPD	MNTD	ses.PD	p	ses.MPD	p	ses.MNDT	p
edible	437	16777	254	47.22	-2.1634	0.017*	-0.334	0.305	-1.190464	0.103
condiment	51	3519.5	257	75.604	-2.7337	0.003*	0.72212	0.746	-3.235228	0.001*
wrapping	26	2115.5	228	101.89	-2.781	0.003*	-1.0441	0.024*	-2.407228	0.006*
cultivated	55	3578.5	253	83.382	-3.4877	0.001*	0.26812	0.565	-2.525447	0.004*
wild	416	16876	253	50.268	1.5522	0.950*	-0.9136	0.127	0.787675	0.785

Table 3. PhyloSor index for estimating turnover and nestedness additive components of the Phylogenetic Beta Diversity (PBD) for the edible species of the Province of the Gulf of Mexico. Comparisons are indicated between wild vs edible, type of use (edible, condiment, wrapping). SES scores >|1.96| are indicated with an asterisk.

Comparisons type of use and management	Nestedness ($p\beta_{sne}$)	True turnover ($p\beta_{sim}$)	Overall ($p\beta_{sim}$)	ses.$p\beta_{sne}$	ses.$p\beta_{sim}$	ses.$p\beta_{sor}$
edible vs condiment	0.17	0.54	0.71	0.21	2.35*	3.44*
edible vs wrapping	0.26	0.57	0.83	-1.49	2.91*	3.53*
condiment vs wrapping	0.53	0.11	0.64	-0.69	2.03*	2.25*
edible vs cultivated	0.03	0.62	0.65	2.64*	1.61	3.55*
edible vs wild	0.05	0.002	0.05	-1.70	1.21	-0.26
condiment vs cultivated	0.46	0.004	0.47	-1.02	0.28	-0.17
condiment vs wild	0.06	0.61	0.67	2.56*	-0.17	2.87*
wrapping vs cultivated	0.66	0.08	0.75	-1.83	3.77*	3.96*
wrapping vs	0.04	0.74	0.78	1.84	0.46	2.82*

wild						
